# *C*-Glycoside metabolism in the gut and in nature: Identification, characterization, structural analyses and distribution of C-C bond-cleaving enzymes

**DOI:** 10.1038/s41467-021-26585-1

**Published:** 2021-11-02

**Authors:** Takahiro Mori, Takuto Kumano, Haibing He, Satomi Watanabe, Miki Senda, Toshio Moriya, Naruhiko Adachi, Sanae Hori, Yuzu Terashita, Masato Kawasaki, Yoshiteru Hashimoto, Takayoshi Awakawa, Toshiya Senda, Ikuro Abe, Michihiko Kobayashi

**Affiliations:** 1 Graduate School of Pharmaceutical Sciences, The University of Tokyo, 7-3-1 Hongo, Bunkyo-ku, Tokyo, 113-0033 Japan; 2 Collaborative Research Institute for Innovative Microbiology, The University of Tokyo, Yayoi 1-1-1, Bunkyo-ku, Tokyo, 113-8657 Japan; 3 PRESTO, Japan Science and Technology Agency, Kawaguchi, Saitama, 332-0012 Japan; 4 Graduate School of Life and Environmental Sciences, University of Tsukuba, 1-1-1 Tennodai, Tsukuba, Ibaraki 305-8572 Japan; 5 Microbiology Research Center for Sustainability, University of Tsukuba, 1-1-1 Tennodai, Tsukuba, Ibaraki 305-8572 Japan; 6 Structural Biology Research Center, Institute of Materials Structure Science, High Energy Accelerator Research Organization (KEK), 1-1 Oho, Tsukuba, Ibaraki 305-0801 Japan; 7 Faculty of Pure and Applied Sciences, University of Tsukuba, 1-1-1, Tennodai, Ibaraki 305-8571 Japan

**Keywords:** Enzyme mechanisms, Enzyme mechanisms, Bacterial structural biology, X-ray crystallography

## Abstract

*C*-Glycosides, in which a sugar moiety is linked via a carbon-carbon (C-C) bond to a non-sugar moiety (aglycone), are found in our food and medicine. The C-C bond is cleaved by intestinal microbes and the resulting aglycones exert various bioactivities. Although the enzymes responsible for the reactions have been identified, their catalytic mechanisms and the generality of the reactions in nature remain to be explored. Here, we present the identification and structural basis for the activation of xenobiotic *C*-glycosides by heterocomplex *C*-deglycosylation enzymes from intestinal and soil bacteria. They are found to be metal-dependent enzymes exhibiting broad substrate specificity toward *C*-glycosides. X-ray crystallographic and cryo-electron microscopic analyses, as well as structure-based mutagenesis, reveal the structural details of these enzymes and the detailed catalytic mechanisms of their remarkable C-C bond cleavage reactions. Furthermore, bioinformatic and biochemical analyses suggest that the *C*-deglycosylation enzymes are widely distributed in the gut, soil, and marine bacteria.

## Abstract (web summary)

In C-glycosides the sugar moiety is linked through a carbon-carbon bond to the non-sugar moiety, which can be cleaved by intestinal microbes. Here, the authors use bioinformatics analysis to identify C-glycoside deglycosidase enzymes in intestinal and soil bacteria, biochemically characterise them and determine their structures and probe catalytic important residues in mutagenesis experiments.

## Introduction

The human gastrointestinal tract contains a complex microbial ecosystem that plays a pivotal role in human health through the maintenance of metabolic homeostasis, regulation of host immunity, and defense against pathogens^1^. In addition to those functions, the transformation of orally ingested xenobiotic chemicals by intestinal microbes is also important for the activation of prodrugs or the inactivation of toxic compounds. However, the molecular mechanisms underlying the activation of bioactive compounds by enzymes in intestinal bacteria remain largely unknown.

Deglycosylation by intestinal microbes is a crucial reaction for the absorption and/or exertion of the biological activities of various compounds^2–4^ that we ingest in food and drugs, as exemplified by the reactivation of drug glucuronide metabolites and the absorption of glycosylated plant-derived bioactive compounds^5–13^. Among several deglycosylation pathways, we focused on the pathway metabolizing *C*-glycosides, of which over 300 kinds have been identified in nature^14^. For example, puerarin and mangiferin have been isolated from the roots of *Pueraria lobata* (“Kudzu”), which is used in traditional Japanese herbal medicine, and from mango peel, respectively. Carminic acid, a *C*-glycoside extracted from cochineal beetles, is used for food staining as a natural “red dye”, over 200 tons being used per year worldwide. Compared with other glycosides (*O*-, *N*- and *S*-glycosides), *C*-glycosides are quite stable against chemical and enzymatic treatments^15^.

**Fig. 1:**
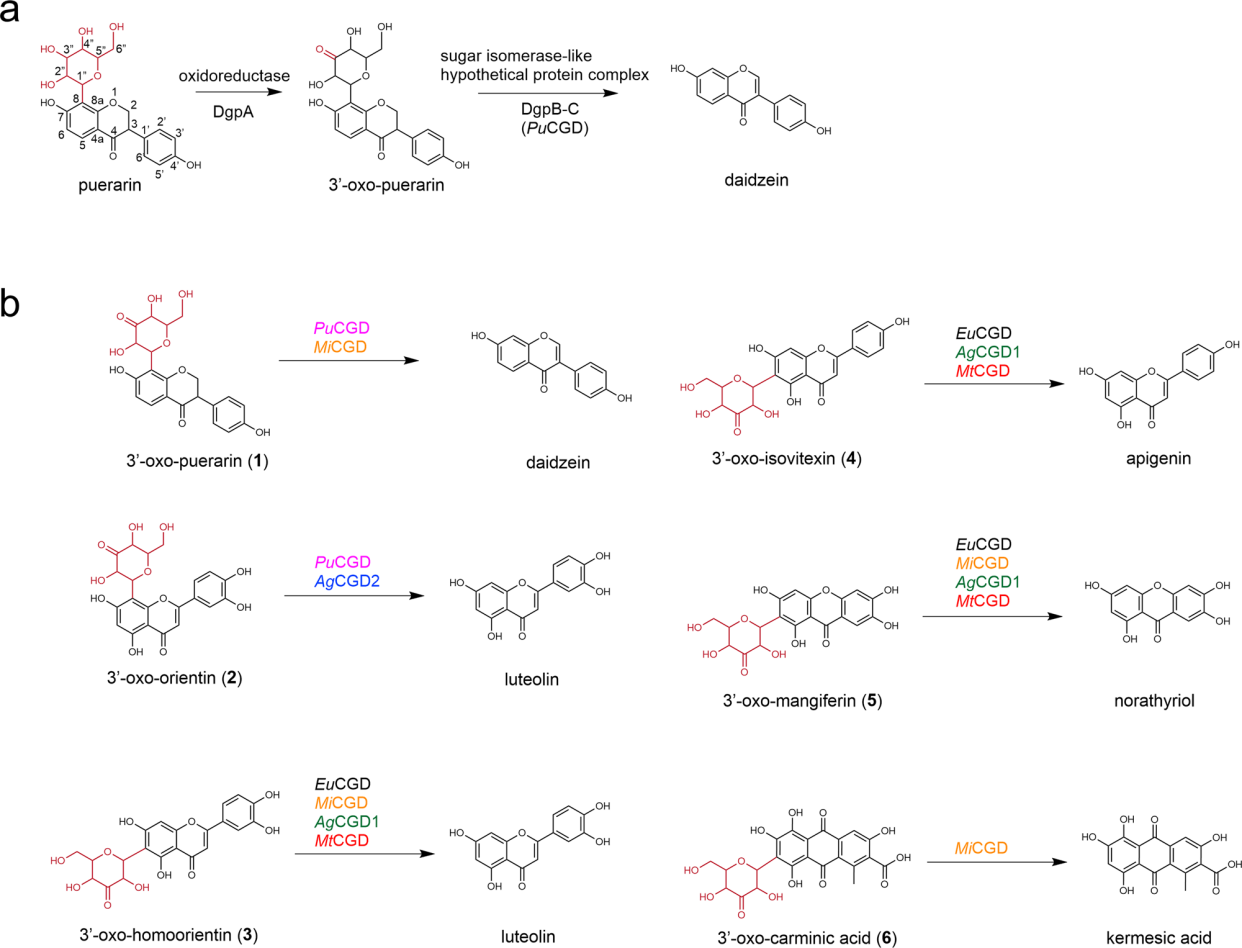
Reactions of *C*-deglycosylation enzymes. **a** Two-step C-C bond cleavage reaction by oxidoreductase (DgpA) and *C*-deglycosylation enzymes (DgpC and DgpB; *Pu*CGD). **b** *C*-deglycosylation enzymes used in our studies: *Pu*CGD, *Eu*CGD, *Mi*CGD, *Ag*CGD1, *Ag*CGD2, and *Mt*CGD. Sugar moieties are shown in red. The substrates investigated in this study were C8-glycosylated flavonoids **1** and **2**, C6-glycosylated flavonoids **3** and **4**, C-glycosylated xanthonoid **5,** and C-glycosylated anthraquinone **6**.

Over the past few decades, several C-C bond cleavage reactions for plant *C*-glycosides due to intestinal bacteria have been reported^5–11,16^. Two metabolic gene clusters, the *dfg* and *dgp* clusters, responsible for the *C*-deglycosylation of flavonoid *C*-glycosides, were recently found in human intestinal bacteria^6,16,17^. DfgA-E in *Eubacterium cellulosolvens* catalyze the deglycosylation of homoorientin and isovitexin^6^, while DgpA (Gfo/Idh/MocA family oxidoreductase), DgpC (α-subunit; sugar isomerase/epimerase-like enzyme), and DgpB (β-subunit; hypothetical protein) in the *dgp* cluster from a PUE bacterial strain catalyze the two-step C-C bond cleavage of puerarin^18^, which shows various pharmacological properties such as antioxidant, anticancer and anti-inflammatory activities, and attenuation of insulin resistance^19^ (Fig. 1a). DgpA catalyzes the oxidation of the glucoside moiety of puerarin to generate 3”-oxo-puerarin, in which the 3”-oxo-sugar moiety is cleaved by an unusual *C*-deglycosylation enzyme complex, which is an α4β4 heterooctamer of DgpB-C, to produce daidzein. DgpC shares less than 20% amino acid sequence similarity with other sugar isomerases^18,20–22^, which do not form a heterocomplex to exert their activities. Although the C-C bond cleavage reactions due to intestinal bacteria are attracting keen interest, the detailed physicochemical properties of the enzymes, their crystal structures, catalytic residues, and catalytic mechanisms, and the generality of the reactions in nature remain unclear.

Here, we describe the X-ray and cryo-EM structures as well as the identification and characterization of *C*-deglycosylation enzymes from intestinal and soil bacteria, and the structural basis of the common C-deglycosylation reaction by the unique enzymes. The generality of the *C*-glycoside-metabolisms in the gut, soil, and marine bacteria was investigated by biochemical and bioinformatic analyses.

## Results

### Phylogenetic analysis of *C*-glycoside deglycosidase (CGD) enzymes

A database search using the amino acid sequences of DgpB and DgpC as queries indicated that both aerobic and anaerobic bacteria, phyla Actinobacteria, Proteobacteria, and Firmicutes, from soil and marine environments and the human digestive system, have each of DgpB and DgpC homologs (Supplementary Fig. 1). As far as we investigated, their gene homologs existed side by side in each bacterial genome (Supplementary Fig. 2 and Supplementary Table 1), suggesting that they form a complex such as DgpB-C to exhibit *C*-glycoside deglycosidase (CGD) activity. Phylogenetic analysis of the homologs revealed that the CGDs share 40-50% amino acid sequence similarity with one another even in different microbial phyla.

### Characterization of intestinal *C*-deglycosylation enzymes

A previous study demonstrated that the combination of five Dfg enzymes, DfgA-E, from *E. cellulosolvens* catalyzed the deglycosylation of the flavone *C*-glycosides homoorientin and isovitexin^6^. Here, sequence analysis revealed that β-galactosidase DfgA and hypothetical protein DfgB share sequence similarity with DgpC and DgpB, respectively (34% and 38% amino acid identities), suggesting that the pair of DfgA and DfgB is involved in the cleavage of the C-C bond (Supplementary Table 1). Expectedly, our studies, such as co-expression, purification, and SEC-MALS analysis of DfgA and DfgB, revealed that DfgA-B formed an α4β4 heterooctamer in solution, as in the case of DgpB-C (Supplementary Fig. 3a and Supplementary Table 2).

**Fig. 2:**
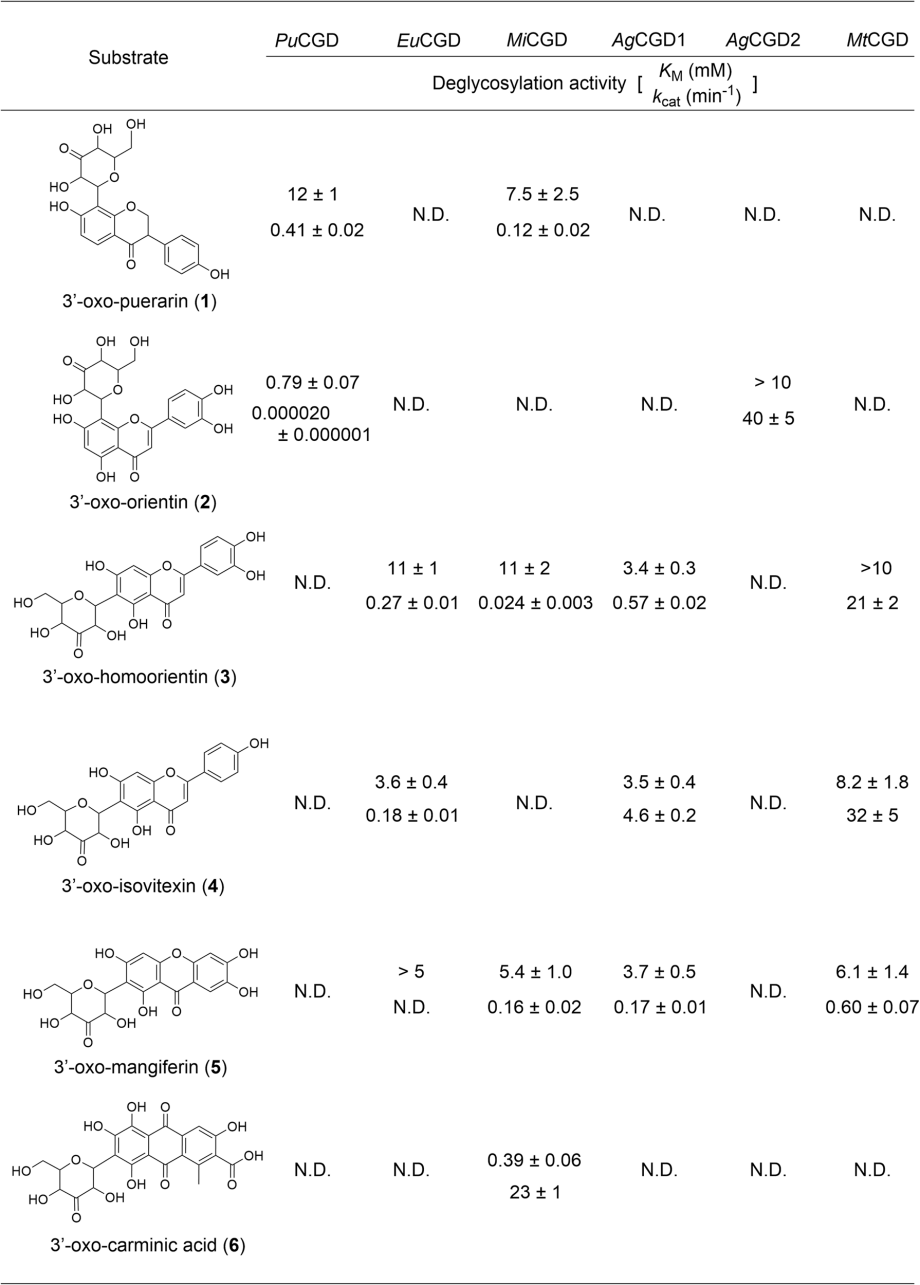
Substrate specificities of *C*-deglycosylation reactions. The kinetic parameters of each *C*-deglycosylation enzyme towards *C*-glycosides were shown. In each set of numbers, the upper and lower numbers represent *K*_M_ (mM) and *k*_cat_ (min^−1^), respectively. N.D.: not determined.

To characterize the biochemical properties of the enzyme complexes in *C*-deglycosylation reactions, the purified DgpB-C and DfgA-B proteins were subjected to in vitro analyses. For clarity, the DgpB-C and DfgA-B complexes are referred to as *Pu*CGD and *Eu*CGD, respectively. First, the metal ion dependency of *Pu*CGD and *Eu*CGD was investigated by the addition of each metal to the chelate reagent-treated enzymes, using 3”-oxo-puerarin or 3”-oxo-homoorientin as a substrate. While the chelate reagent-treated *Pu*CGD and *Eu*CGD showed activity reduced to 16% and 37% as compared to the non-chelate reagent-treated enzyme (represented as WT in Supplementary Fig. 4a), respectively, the activity was restored by the addition of divalent metal ions other than Cu^2+^ and Fe^2+^. The addition of Ni^2+^ and Mn^2+^ to each of *Pu*CGD and *Eu*CGD improved their activities up to 157% and 152% (for *Pu*CGD) and 131% and 112% (for *Eu*CGD), respectively. (Supplementary Fig. 4). These findings indicated that each of the divalent metal ions facilitated the C-C bond cleavage reaction. The optimal pH and temperature of both *Pu*CGD and *Eu*CGD were pH 6.0 and 60 °C (Supplementary Fig. 4b and c). To elucidate the substrate specificity of these enzymes, each of 3”-oxo-C6- or C8-glycosylated flavonoids, 3’-oxo-carminic acid and 3’-oxo-mangiferin **1**-**6**, was incubated with *Pu*CGD and *Eu*CGD (Figs. 1, 2, and Supplementary Fig. 5). As a result, *Pu*CGD accepted the C8-glycosylated compounds **1** and **2**, but the C6-glycosylated compounds **3**-**6** were inert substrates. In contrast, *Eu*CGD acted on C6-glycosylated compounds **3**-**5**, but not on C8-glycosylated compounds **1** and **2**. The steady-state kinetics values for deglycosylation reactions by *Pu*CGD were *K*_M_ = 12 mM and *k*_cat_ = 0.41 /min for **1** and *K*_M_ = 0.79 mM and *k*_cat_ = 0.020 × 10^−3^ /min for **2**. On the other hand, the steady-state kinetics values for deglycosylation reactions by *Eu*CGD were *K*_M_ = 11 mM and *k*_cat_ = 0.27 /min for **3** and *K*_M_ = 3.6 mM and *k*_cat_ = 0.18 /min for **4** (Fig. 2).

### Identification of the *C*-deglycosylation enzyme from soil bacteria

As described above, phylogenetic analysis of and a database search for *Pu*CGD and homologs suggested wide distribution of CGDs in nature. Indeed, in the genome of *Microbacterium* sp. 5-2b, which we recently discovered as a *C*-glycoside carminic acid-metabolizing bacteria on assimilation screening of a soil environment, gene products sharing 29/49% and 30/52% amino acid identity/similarity with the α- and β-subunits of *Pu*CGD were found and designated as CarB and CarC, respectively (Supplementary Fig. 2 and Supplementary Table 1). No other gene products in *Microbacterium* sp. 5-2b showed similarity to *Pu*CGD. To examine the universality of *C*-deglycosylation reactions and their biochemical functions, CarΒ and CarC were expressed individually and incubated with 3’-oxo-carminic acid as a substrate (Supplementary Fig. 6). On incubation of CarB and CarC together, 3’-oxo-carminic acid was converted to kermesic acid, although neither CarB nor CarC showed 3’-oxo-carminic acid-converting activity (Supplementary Fig. 7). We next co-expressed His-tagged CarB and tag-free CarC. As a result, His-tagged CarB was purified together with CarC by using a Ni-affinity column (Supplementary Fig. 3b). SEC-MALS analysis revealed that the molecular mass of the CarB-C complex was 49.0 kDa. Together with the theoretical masses of CarB (39.4 kDa) and CarC (19.6 kDa) calculated from their deduced amino acid sequences, these findings indicated that CarB and CarC formed a heterodimer, while *Pu*CGD and *Eu*CGD formed an α4β4 heterooctamer. We here designated CarB and CarC as the α-subunit and β-subunit of *Mi*CGD, respectively. The purified *Mi*CGD showed the catalytic activity converting 3’-oxo-carminic acid to kermesic acid without any cofactors as in the case of *Pu*CGD and *Eu*CGD (Fig. 1b). The absorption spectrum of the purified *Mi*CGD showed an absorbance maximum near 280 nm. No other absorption peak or shoulder was observed at higher wavelengths (Supplementary Fig. 6c). These results suggested that no cofactor which exhibits UV absorption was bound to the purified *Mi*CGD enzyme.

A BLAST search identified more putative CGD homologs in soil bacteria. Two homologs from *Arthrobacter globiformis*, and one from *Microbacterium trichothecenolyticum* were designated as *Ag*CGD1, *Ag*CGD2, and *Mt*CGD, respectively, and heterologously expressed in *E. coli* (Supplementary Figs. 1a, 2, and 3b, and Supplementary Table 1). The SEC-MALS analysis revealed that the purified enzymes of soil bacteria were αβ heterodimers like *Mi*CGD (Supplementary Table 2). The optimal temperatures of soil bacterial CGDs, including *Mi*CGD, *Ag*CGD1, *Ag*CGD2, and *Mt*CGD, were around 40 °C (Supplementary Fig. 4d). The optimal pH for the activities of *Mi*CGD and *Ag*CGD1 was pH 7.5, and that for *Mt*CGD was pH 6.0 (Supplementary Fig. 4e).

Substrate specificity analysis of the soil bacteria-derived enzymes as to compounds **1**-**6** demonstrated that they also catalyzed the C-C bond cleavage of various *C*-glycosylated compounds (Figs. 1b, 2, and Supplementary Fig. 8). *Mi*CGD showed high activity toward **6** and weakly accepted **1** and **3**-**5**. *Ag*CGD1 and *Mt*CGD showed activity toward **3**-**5**, while *Ag*CGD2 only accepted **2** as a substrate. These results indicated that *Mi*CGD, *Ag*CGD1 and *Mt*CGD mainly accepted the C6-glycosylated compounds as substrates, while *Ag*CGD2 accepted the C8-glycosylated compound. The steady-state kinetics values for deglycosylation reactions by those enzymes were calculated (Fig. 2). The *K*_M_ value of *Mi*CGD for **6** was 0.39 mM. On the other hand, most *K*_M_ values were around 3 to 10 mM.

Similar to the intestinal enzymes (*Pu*CGD and *Eu*CGD), after dialysis with chelating agents, the enzymatic activities of *Mi*CGD, *Ag*CGD1, and *Ag*CGD2 were reduced to 13, 1.4, and 0%, respectively. As compared with the non-dialyzed enzyme (denoted as WT in Supplementary Fig. 4a), the activities of *Mi*CGD and *Ag*CGD1 were restored to 124% and 104%, respectively, by adding Mg^2+^. On the other hand, the activity of *Ag*CGD2 was restored by the addition of each of Ni^2+^, Mn^2+^, Ca^2+^, Mg^2+^, and Co^2+^ up to 55%, 119%, 84%, 90%, and 87%, respectively (Supplementary Fig. 4a).

### X-ray crystal structures of *C*-deglycosylation enzyme complexes

**Fig. 3:**
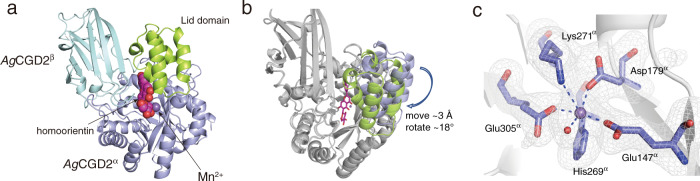
Crystal structures of *C*-deglycosylation enzymes. **a** Heterodimer structure of *Ag*CGD2. The β-sandwich structure of *Ag*CGD2^β^ is shown in pale blue and the TIM barrel structure of *Ag*CGD2^α^ in purple. The lid domain of *Ag*CGD2^α^ is shown in light green. **b** Conformational change of the lid domain of *Ag*CGD2^α^ upon substrate binding. The lid domains in the apo structure and the structure complexed with homoorientin are shown in purple and light green, respectively. **c** The Fo-Fc polder omits the map of the metal-binding site of *Ag*CGD2^α^. The electron density map of the ligands is represented by a gray mesh, contoured at +3.0 sigma. Purple and red balls represent Mn^2+^ and water, respectively.

To elucidate the molecular basis of the enzymatic C-C bond cleavage reaction in the activation/inactivation of xenobiotic compounds by CGDs, we solved the apo structures of *Pu*CGD, *Eu*CGD, and *Ag*CGD2, and the co-substrate structure of *Ag*CGD2 with homoorientin, at 2.50, 2.40, 2.30 and 2.25 Å resolution, respectively (Fig. 3, Supplementary Fig. 9a and b and Supplementary Table 3). While these three CGDs shared similar heterodimer unit structures, *Pu*CGD and *Eu*CGD formed α4β4 heterooctamers and *Ag*CGD2 formed an αβ heterodimer (Fig. 3, and Supplementary Fig. 9a and b). And Fig. 3a showed that homoorientin bound in a cavity at the interface between the α- and β-subunits of *Ag*CGD2, indicating that the active site was created through heterodimerization.

Each β-subunit of both enzymes consisted of seven parallel β-sheets and formed a β-sandwich fold (Fig. 3a and Supplementary Fig. 9a and b). In contrast, the overall structure of *Pu*CGD^α^ (which represents the α-subunit of *Pu*CGD) adopted a TIM-barrel fold, consisting of nine parallel β-strands assembled into a circular β-barrel, surrounded by a ring of solvent-exposed α-helices. A Dali search revealed that the *Pu*CGD^α^ structure exhibited moderate similarities with those of known sugar isomerases, including the xylose isomerase from *Planctopirus limnophila* (PDB ID: 4OVX) and the sugar-phosphate isomerase/epimerase from *Parabacteroides distasonis* ATCC 8503 (PDB ID: 3P6L), with Z-scores of 27.4 and 23.6, and RMSD values of 2.6 and 2.3 Å for the 270 and 262 Cα-atoms, respectively (19 and 17% amino acid sequence identities, respectively).

The heterodimer structures of *Eu*CGD and *Ag*CGD2 were similar to that of *Pu*CGD, with RMSD values of 2.2 and 3.5 Å, respectively (Fig. 3a–c, and Supplementary Fig. 9a and b). The interaction angle of *Ag*CGD2^α^ and *Ag*CGD2^β^ was clearly different from that of *Pu*CGD, while the *Ag*CGD2^α^ structure was almost identical to that of *Pu*CGD^α^.

The major structural differences among these enzymes lay on the interface between the α- and β-subunits; the α-subunits of *Pu*CGD and *Ag*CGD2 (represented as *Pu*CGD^α^ and *Ag*CGD2^α^, respectively) were composed of a TIM barrel domain and an additional domain with four α-helices (lid domain), which was not observed in *Eu*CGD^α^ (Fig. 3a and Supplementary Fig. 9a and b). Instead, the loop between M121-N133 of *Eu*CGD^α^, forming the active site cavity, was six residues longer than those of *Pu*CGD^α^ and *Ag*CGD2^α^. Moreover, the *N*-terminal loops of *Eu*CGD^β^ and *Ag*CGD2^β^ were also short as compared to that of *Pu*CGD^β^. The loop of *Pu*CGD^β^ formed the entrance of the cavity, while the corresponding loops of *Eu*CGD^β^ and *Ag*CGD2^β^ extended in different directions (Fig. 3a–c, and Supplementary Fig. 9a and b). On the other hand, 11 residues were inserted in the loop between β7 and β8 of *Eu*CGD^β^ (T110-V139), as compared to those of *Pu*CGD^β^ and *Ag*CGD2^β^, and the loop was involved in the formation of the active site cavity.

### Cryo-EM structures of *C*-deglycosylation enzyme complexes

**Fig. 4:**
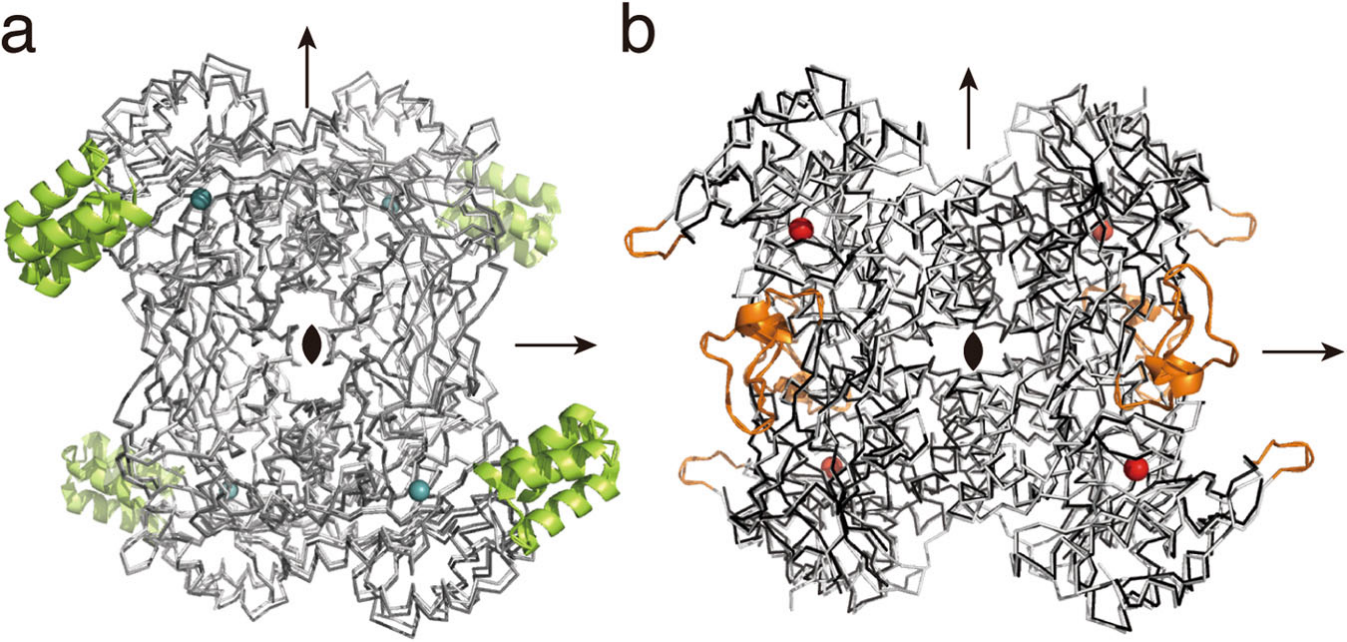
Comparison of overall structures between X-ray crystal structures and cryo-EM structures. Comparison of X-ray crystal structures (pale gray) and cryo-EM structures (gray) of (**a**) *Pu*CGD and (**b**) *Eu*CGD (front view). Missing residues in cryo-EM structures are shown in (**a**) lime and (**b**) orange. Arrows represent approximate 2-fold axes.

To obtain more structural details of *C*-deglycosylation enzymes, and to examine the flexibility of the lid-domain and the loop regions in *Pu*CGD and *Eu*CGD, we also determined the structures of *Pu*CGD and *Eu*CGD at 2.85 and 2.54 Å resolution, respectively, using single-particle cryo-EM (Supplementary Fig. 10 and Supplementary Table 4). The α4β4 heterooctamer structures of both enzymes were almost identical to those of the X-ray crystal structures (RMSD values 0.6 and 0.4 Å, respectively) (Fig. 4). Intriguingly, however, the densities of the lid domain in *Pu*CGD^α^ and the long loops in *Eu*CGD were disordered in the cryo-EM structures. These results suggested that open-to-closed conformational changes of the lid domain occurred upon substrate binding. Moreover, a comparison between the crystal structures of apo *Ag*CGD2 and its homoorientin complex revealed that the lid domain of *Ag*CGD2 moved ~3 Å and rotated ~18 degrees toward the active site upon substrate binding (Fig. 3b).

Truncation of the lid domain of *Pu*CGD^α^ completely abolished the C-C bond cleavage activity, but did not affect protein expression or α4β4 heterooctamer formation (Supplementary Fig. 3a and c). Although the *Eu*CGD complex did not possess a lid domain, the disordered loop regions would play a similar role in the active site formation (Fig. 4b).

### Active site architecture

**Fig. 5:**
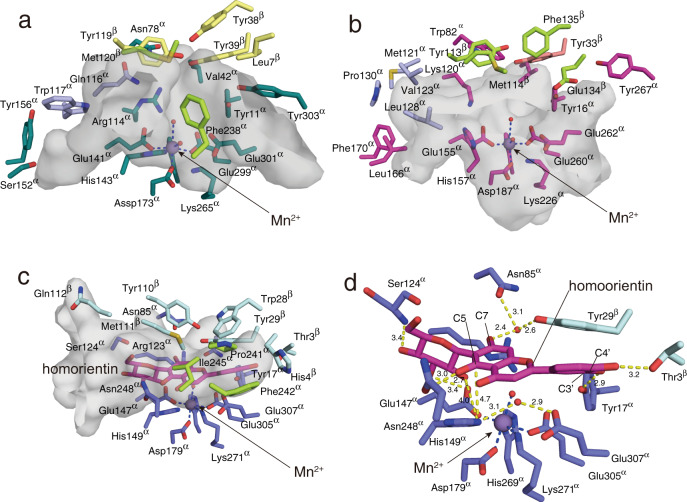
Comparison of the active site architectures of *C*-deglycosylation enzymes. **a**–**c** Comparison of the active sites of (**a**) *Pu*CGD, (**b**) *Eu*CGD, and (**c**) *Ag*CGD2 with homoorientin (colored in magenta). Side views of the active site cavities are depicted as gray surfaces. **d** Interactions between homoorientin and active site residues in *Ag*CGD2. The hydrogen bonds are shown by dashed yellow lines.

**Fig. 6:**
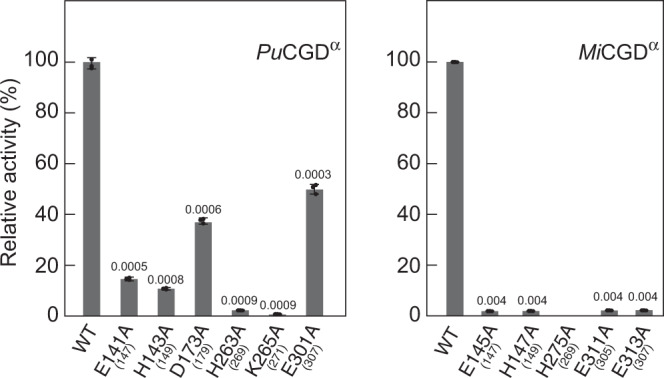
Mutagenesis analysis of *Pu*CGD^α^ and *Mi*CGD^α^. The numbers in brackets represent the corresponding numbers of the residues in *Ag*CGD2. All experiments were repeated independently more than three times, and similar results were obtained. Statistical comparisons between means for the wild type (WT) and each mutant were performed by Student’s t-test (2-tailed). The *p*-values were shown above each bar. The bars are means of *n* = 3 independent experiments (each data point was indicated by a black circle) and error bars indicate standard deviations. Data are presented as mean values + /− SD. All experiments were repeated independently three times with similar results.

The crystal structure of *Ag*CGD2 complexed with homoorientin (C6-glycosylated flavone) indicated that the active site was located between the α- and β-subunits. The difference anomalous Fourier maps of *Ag*CGD2, *Pu*CGD, and *Eu*CGD suggested that these enzymes contain Mn^2+^ as a metal ion in the active site (Supplementary Fig. 11). The manganese ion was coordinated by Glu147^α^ (which represents Glu147 in the α-subunit of *Ag*CGD2), Asp179^α^, His269^α^, Lys271^α^, Glu305^α^, and a water molecule (Figs. 3c and 5). In the *Pu*CGD structure, although a water molecule coordinated to the metal ion instead of Lys271^α^, the positions of the bound metal and the residues 1His/2Glu/1Asp/1Lys were well conserved in the three homologs (Figs. 3c and 5, and Supplementary Fig. 9c and d). Notably, the mutations of these residues to alanine in *Pu*CGD and *Mi*CGD (E147^α^A, D179^α^A, H269^α^A, K271^α^A, and E305^α^A mutants [the numbering of each of which follows the residues in *Ag*CGD2]) dramatically reduced the C-C bond cleavage activity, indicating that metal-binding was essential for the enzyme reaction (Fig. 6). Although the CGDs accepted various metals to exhibit their activities, these observations suggested that the enzymes utilize Mn^2+^ as a native metal ion. On the other hand, the occupancy of the metal ion was relatively low. Considering the results of the metal dependency experiment showing that chelate reagent-treated enzymes exhibited higher activities on the addition of the metal ion than non-treated enzymes, some CGDs lacked metals in the heterologous expression or purification steps. This partial metal occupancy may affect enzyme activity as a result and cause small errors in the coordination sphere of the metal ions in the crystal structure.

While the metal-coordinating residues were only in the α-subunit, the aglycone moiety of homoorientin interacted with the residues in both subunits; for example, the hydroxyl groups at C3’, C4’, and C5 interacted with Thr3^β^, Tyr17^α^, and Asn248^α^, respectively, and the hydroxyl groups at C2” and C3” of the sugar moiety interacted with Glu147^α^, and the hydroxyl group at C4” interacted with Ser124^α^ (Fig. 5d). On the other hand, the docking model of *Ag*CGD2 with orientin (C8-glycosylated flavone), constructed based on the structure of a complex with homoorientin, suggested that the active site pocket of *Ag*CGD2 is large enough to accommodate orientin with a similar sugar-binding mode (Supplementary Fig. 12a and b). In this model, the C7 and C3’ of the aglycon moiety interacted with Arg123^α^ and the mainchain of Gln112^β^, respectively.

The structure of *Ag*CGD2 complexed with homoorientin indicated that the aglycon moiety was firmly fixed through a hydrogen-bonding interaction and surrounded by hydrophobic residues in the active site cavity, and that there were no space to change the conformation of the aglycon part for C6-glycosides; the conformational change would be necessary for this reaction to proceed, because the aglycon is dearomatized and the C6 or C8 carbon atom, which connects with glycoside, is changed to an sp^3^ from an sp^2^ carbon in the enzyme reaction. In contrast, the docking model of *Ag*CGD2 with orientin suggested that the enzyme possessed enough space for alteration of the conformation in the active site for C8-glucosides. These would be the possible reasons why *Ag*CGD2 acted on C8-glycosylated compounds, rather than C6-glycosylated ones.

Sequence alignment among *Ag*CGD2, *Mi*CGD, *Ag*CGD1, and *Mt*CGD revealed that the residues that interact with hydroxyl groups of the aglycon of homoorientin in the *Ag*CGD2 structure (including Tyr17^α^ and Asn248^α^) are well conserved (Supplementary Fig. 13). On the other hand, the loop in *Ag*CGD2^α^ comprising Pro241^α^ and Phe242^α^ is shortened, and Met249^α^, which formed the active site cavity in *Ag*CGD2, is substituted with a small amino acid, Ala or Gly, in the other enzymes. Moreover, the loop region between β7 and β8 (R107-G120 in *Ag*CGD2^β^), which is located close to the aglycon moiety, was not conserved and four residues were found to be inserted in *Mi*CGD^β^, *Ag*CGD1^β^, and *Mt*CGD^β^ (Supplementary Fig. 13). These differences would alter the shape of the active site and allow *Mi*CGD, *Ag*CGD1, and *Mt*CGD to act on C6-glycosides rather than C8-glycosylated compounds.

We also constructed docking models of *Pu*CGD and *Eu*CGD with orientin and homoorientin, based on the co-crystal structure of *Ag*CGD2 with homoorientin, to determine the structural basis of the substrate specificity of these enzymes (Supplementary Fig. 12c-f). The models suggested that *Pu*CGD and *Eu*CGD possess significant space to accept orientin and homoorientin, respectively (Supplementary Fig. 12d and e). On the contrary, *Pu*CGD did not accept homoorientin because Tyr303^α^ and Leu7^β^ clash with the B-ring of homoorientin in the model (Supplementary Fig. 12c). In the model of *Eu*CGD with orientin, furthermore, the B-ring of orientin also clashed with active site residues including Leu128^α^ and Pro115^β^ (Supplementary Fig. 12f). These observations are consistent with the substrate specificities of *Pu*CGD and *Eu*CGD (Figs. 1 and 2).

As described above, the active site was formed by the amino acid residues from each subunit: the α-subunit bound metal, which was essential for the reaction, and the β-subunit was involved in the substrate binding. To investigate whether the α- and β-subunits of each *C*-deglycosylation enzyme complex are exchangeable, we performed a swapping experiment on the β-subunits to construct *Pu*CGD^α^-*Eu*CGD^β^ and *Eu*CGD^α^-*Pu*CGD^β^. The pull-down assays revealed that the β-subunits were not co-eluted with the α-subunits in both cases, indicating that the subunits of each *C*-deglycosidase were not exchangeable for the heterodimer formation (Supplementary Fig. 3d); in other words, the complex structure of each enzyme was crucial for its specific enzymatic activity.

### Identification of catalytic residues

It has been proposed that 3”-oxo-puerarin is nonenzymatically isomerized to the 2”-oxo form in a basic buffer, to generate 2”-ene-,3”-diol-puerarin as a putative intermediate^16,17^. Considering the slightly acidic environment of a typical human gastrointestinal tract, and the optimal pH of the *Pu*CGD and *Eu*CGD enzyme reactions, however, one or more basic residues could facilitate the deprotonation from the C2” position of the sugar moiety and the dearomatization of the A-ring in the flavonoid to catalyze the C-C bond cleavage reaction. Indeed, conserved His149^α^ (in *Ag*CGD2) is situated near the homoorientin in *Ag*CGD2, suggesting that His149^α^ can be involved in the catalytic reaction. The crystal structure of *Ag*CGD2 in complex with homoorientin showed that distances of ND_His149_–C2”_homoorientin_ and NE_His149_–C6_homoorientin_ were 4.2 Å and 4.7 Å, respectively (Supplementary Fig. 12a and b). Moreover, the conserved Glu307^α^ interacted with His149^α^ via a water molecule in the active site of *Ag*CGD2. A Similar hydrogen bond network was also observed in the active site of *Pu*CGD. Notably, the docking model of *Ag*CGD2 with orientin (C8-glycosylated flavone) suggested that the ND atom of His149^α^ was located at a distance of 3.8 Å from the C2” atom of orientin, which is close enough to abstract the C2” hydrogen atom. Furthermore, a water molecule coordinating to the metal ion was positioned close to the C7 hydroxyl group at a distance of 3.5 Å (Supplementary Fig. 12b).

To investigate the roles of these residues, therefore, the conserved His and Glu residues in the C8-glycoside-specific *Pu*CGD and the C6-glycoside-specific *Mi*CGD were substituted with Ala. The enzyme reaction with 3”-oxo-puerarin revealed that the activities of the H143^α^A and E301^α^A mutants of *Pu*CGD (which corresponded to H149 and E307 in *Ag*CGD2, respectively) were reduced to 10.4% and 49.8%, respectively (Fig. 6). Furthermore, the H147^α^A and E313^α^A mutants of *Mi*CGD showed dramatically reduced C-C bond cleavage activity (to 1.8 and 1.9%, respectively), as shown in Fig. 6. These findings suggested that the histidine residue facilitated the reaction as a base catalyst to deprotonate the C5-hydroxyl group of the aglycone and the C2 position of the sugar moiety via a water molecule. Meanwhile, the glutamic acid residue would support the positioning of the water molecule through hydrogen bond interactions.

## Discussion

Microorganisms are involved in the degradation of natural and some artificial compounds, the resultant material resources being returned for use in nature^23^. However, only limited numbers of metabolic pathways for various natural or artificial compounds have been identified. In previous studies, we identified the novel microbial metabolism of and metabolic enzymes for artificial compounds such as nitrile^24^ and isonitrile^25^, and natural compounds such as sesamin and curcumin^26,27^.

Glycosides are ubiquitous compounds in nature. Plants synthesize glycosylated flavonoids, which are transferred to vacuoles or the cell wall^28^, and microorganisms synthesize glycosylated antibiotics such as erythromycin^29^, vancomycin^30^, lankamycin^31^, lyncomycin^32^, and avermectin^33^. We daily ingest plant-derived glycosylated compounds from vegetables and fruit. Those glycosides have been reported to be metabolized by gut microbes in the large intestine^5^; the resulting aglycones are absorbed from the intestine and show various bioactivities in our body^34^. While a lot of microbes catalyzing *C*-glycoside deglycosylation reactions for various flavonoids and terpenoids have been reported^5–11^, to the best of our knowledge, all of the so far known microbes were intestinal bacteria and the catalytic mechanisms for the reaction (involving the combination of active amino acid residues and a metal ion) remain unclear. In organic chemistry, on the other hand, C–C bond cleavage is a large issue because of its inert property. Although some strategies such as the use of transition-metals or generation of radicals have enabled to activate a C-C bond^35^, the cleavage of the inherently inert bond is still challenging.

In this study, we characterized two *C*-glycoside deglycosylation enzymes (CGDs) from intestinal bacteria and three CGDs from soil bacteria, which were isolated through assimilation screening and genome mining. Further detailed biochemical analysis of CGDs from intestinal and soil bacteria revealed that these enzymes catalyzed selective C-C bond cleavage reactions toward 3’-oxo-C6- or C8-glycosylated compounds, while they exhibited broad substrate specificity toward the aglycone structures. Furthermore, SEC-MALS analysis revealed CGDs from intestinal bacteria were α4β4 heterooctamer proteins, while those from soil bacteria were αβ heterodimer proteins, which could be the reason why the optimal temperatures of intestinal bacteria (60 °C) were higher than those of soil bacteria (40 °C, Supplementary Fig. 4). These differences in subunit structures may be due to the difference in the bacterial phyla: *Microbacterium* and *Arthrobacter* belong to the phylum Actinomycetes, while intestinal *E. cellulosolvens* and the PUE strain belong to the phylum Firmicutes.

**Fig. 7:**
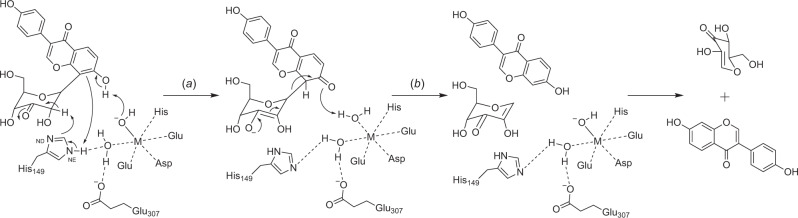
Proposed mechanism of *C*-deglycosylation reaction. The numbering of amino acid residues in this figure is that for *Ag*CGD2. M represents a metal ion.

Through the crystallographic analysis of CGDs, we identified the relationships between the enzyme structure and function of CGDs. (i) The active site of CGD was formed by the interface between the α- and β-subunits and the complex structure of each enzyme was crucial for enzymatic activity. (ii) The conformational change of the lid domain would be important for the formation of the active site for catalysis. (iii) The metal ion was bound in the α-subunit, while several residues in the β-subunit were involved in the binding of a substrate. The effects of chelating agents and site-directed mutagenesis analysis revealed that metal-binding was essential for the enzyme reaction. (iv) The shape of the active site was determined by the combination of α- and β-subunits, which plays a critical role in the substrate specificities of CGDs. Based on the crystal structures together with the results of biochemical and site-directed mutagenesis analyses, we propose a detailed mechanism for the C-C bond cleavage reactions of the unique *C*-deglycosylation enzyme complexes, as follows (Fig. 7 and Supplementary Fig. 14). (a) After the substrate binds to the active site with a conformational change of the lid domain, and the sugar moiety binds close to the coordinated metal and His149^α^, a metal-hydroxide ion abstracts a proton from the hydroxyl group at the ortho or para position of the glycosylated carbon in the aglycone of the substrate. And then deprotonation of NE of His149^α^ occurs to dearomatize the aglycone moiety, followed by abstraction of a hydrogen atom from the C2” position of the glycoside by His149^α^ as a base catalyst to form the 2”-ene-2”, 3”-diol intermediate. The hydrogen bond interactions among His149^α^, Glu307^α^, and a water molecule, and deprotonated NE of His149^α^ could facilitate the abstraction of the C2” hydrogen atom. (b) The C-C bond between the aromatic ring and the sugar moiety is cleaved through a β-elimination-like reaction, to generate 1,5-anhydro-D-erythro-hex-1-en-3-ulose and the aglycone.

While C-C bond cleavage reactions have been reported for the biosynthesis or metabolism of natural products such as steroids, lignans, and fatty acids, these reactions are catalyzed by oxidative enzymes, including P450, radical SAM enzyme, and flavoproteins^36^. In contrast, although the *C*-deglycosylation reaction is also initiated by oxidation of the C3-position of a glycoside by oxidoreductase, the C-C bond cleavage reaction of CGDs is a metal-assisted general acid/base mechanism. *O*-deglycosylation with a β-elimination-like reaction has been observed in the reactions of some glycoside hydrolase enzyme families^37^, which catalyze the oxidation of the C3-hydroxyl groups of glycosides, followed by a nonenzymatic β-elimination-like reaction to cleave the C-O bonds in *O*-glycosides. In contrast, the cleavage of the inert C-C bond shown here requires *Pu*CGD homologs after C3-oxidation by oxidoreductase, indicating the importance of the dearomatization and subsequent deprotonation steps for the C-C bond cleavage by the unique *C*-deglycosylation enzyme complexes.

As described above, CGDs and microbes that perform bioconversion of *C*-deglycoside compounds to aglycone have been identified in only intestinal bacteria^18^. However, together with our microbial screening, a database search suggested that CGD homologs are widely distributed in various bacterial phyla (Supplementary Fig. 1), indicating the universality of rare C-C bond cleavage reactions in nature. Considering that we identified *Mi*CGD from a *C*-glycoside-catabolizing microorganism on assimilation screening, the biological function of the *C*-deglycosylation reaction would be involved in the uptake of sugar as a carbon source from natural glycosylated compounds. Further identification of *C*-deglycosylation enzymes and investigation of the functions of other enzymes in the gene cluster will provide insights into the metabolic cycle of glycosylated compounds.

In conclusion, our structure-function analysis of *C*-deglycosylation enzymes revealed the overall structures, active site architecture, and key structural changes. Based on these structural observations and mutagenesis experiments, the C-C bond cleavage mechanism involving acid/base catalysis was proposed. We also indicated the generality of the reaction in both soil and intestinal microorganisms through biochemical and structural studies. Because the C-C bond cleavage is crucial for *C*-glycosides to exert their biological activities, these findings will facilitate clarification of the bio-availability of xenobiotic *C*-glycosides in humans and the biogeochemical circulation of *C*-glycosides in nature. Future biochemical and structural analyses of the enzymes from intestinal bacteria will provide further insights into the molecular basis of the activation of prodrugs.

## Methods

### General remarks

Oligonucleotide primers were purchased from Eurofins Genomics. Other chemicals were purchased from Wako Chemical Ltd., and Kanto Chemical Co. Inc., (Tokyo, Japan). The LCMS data were obtained using a compact microTOF-MS (Bruker) attached to an LC-20AD UHPLC system (Shimadzu) with a COSMOSIL 2.5C18-MS-II column (2 mm i.d. × 75 mm; Nacalai Tesque, Inc.). Analytical HPLC was performed on a Shimadzu LC20-AD HPLC system, using a Thermo Scientific Hypersil GOLD analytical column (4.6 ×250 mm, 5 μm). 3-Oxo-glucose and 3”-oxo-puerarin were synthesized according to the published method^17^.

### Cloning and heterologous expression of DgpA, DfgE, *Pu*CGD, *Eu*CGD, *Mi*CGD*, Ag*CGD1, *Ag*CGD2, and *Mt*CGD

DNA fragments encoding DgpA, DgpB, DgpC, DfgA, DfgB, and DfgE were synthesized after codon optimization for expression in *E. coli* by FASMAC Co., Ltd (Supplementary Table 6). The full length of each gene was amplified using the corresponding primers listed in Supplementary Table 5. For DgpA, *Pu*CGD, and *Eu*CGD, each PCR product was cloned into the linearized pET22b vector (Merck Millipore) for expression as fusion proteins with a C-terminal His_6_-tag. For the construction of the *Pu*CGD and *Eu*CGD plasmids, the amplified DgpB and DgpC fragments were tandemly ligated into the linearized pET22b vector. The amplified DfgE was inserted into the digested pET28a-MBP vector. For *Mi*CGD*, Ag*CGD1, *Ag*CGD2, and *Mt*CGD, each PCR product was cloned into the linearized pET24a(+) vector by the In-Fusion protocol (Clontech Laboratories, Inc.).

After sequence confirmation, the vectors containing the target genes were transformed into *E. coli* BL21(DE3) competent cells (in the case of DgpA, DfgE, *Pu*CGD, and *Eu*CGD) or *E. coli* Rosetta2 (DE3) cells (in the case of *Mi*CGD*, Ag*CGD1, *Ag*CGD2, and *Mt*CGD). The cells harboring the plasmids were cultured at 37 °C in LB medium, containing 100 μg ml^−1^ ampicillin (DgpA, DfgE, *Pu*CGD, and *Eu*CGD) or 2×YT media containing 50 μg/ml kanamycin and 30 μg/ml chloramphenicol (*Mi*CGD*, Ag*CGD1, *Ag*CGD2, and *Mt*CGD). To express the target proteins, isopropyl β-D-thiogalactopyranoside (IPTG) was then added to 0.2 mM (DgpA, DfgE, *Pu*CGD, and *Eu*CGD) or 0.5 mM (*Mi*CGD*, Ag*CGD1, *Ag*CGD2, and *Mt*CGD) (final concentration) when the OD_600_ reached 0.6, and the cultures were continued for 20 h at 16 °C (DgpA, DfgE, *Pu*CGD, and *Eu*CGD) or 18 °C (*Mi*CGD*, Ag*CGD1, *Ag*CGD2, and *Mt*CGD). All of the following procedures were conducted at 4 °C.

For purification of DgpA, *Pu*CGD, and *Eu*CGD, the cultured cells were harvested by centrifugation at 6,000 × g and resuspended in 50 mM HEPES (pH 7.6), containing 300 mM NaCl, 10% glycerol, and 10 mM imidazole (lysis buffer). For cell lysis, 1 mg ml^−1^ lysozyme was added to the solution, followed by incubation at 4 °C for 1 h with slow rotation. The cells were then further disrupted by sonication on ice. The insoluble debris was removed by centrifugation at 12,000 × g for 45 min. The supernatant was loaded onto a HisPur^TM^ Ni-NTA Resin (Thermo Fisher Scientific) column, which was then washed with 50-column volumes (CVs) of lysis buffer containing 20 mM imidazole. Then, the enzymes were eluted with 3 CVs of lysis buffer containing 500 mM imidazole. The purified proteins were then applied to a Resource Q column and the bound proteins were eluted with a linear gradient of 0.05–1 M NaCl in 50 mM HEPES buffer (pH 7.6). The pooled protein from the Resource Q column was further purified by gel-filtration chromatography on a Superose 6 column (GE Healthcare), which was eluted with 20 mM HEPES (pH 7.6) buffer containing 100 mM NaCl. The concentration of each protein was calculated by measuring the ultraviolet absorption at A_280_^38^.

For the purification of *Mi*CGD*, Ag*CGD1, *Ag*CGD2, and *Mt*CGD, the pellet (20 g) was resuspended in twenty milliliters of 20 mM Tris-HCl buffer (pH 8.0). The cells were disrupted by sonication, as described above. The lysate was centrifuged at 27,000 × *g* at 4 ˚C for 20 min. The recombinant proteins were purified using a His Trap HP column (GE Healthcare).

For the purification of DfgE, after His-tag purification, the purified fusion protein was dialyzed for 16 h in cold buffer containing 50 mM HEPES (pH 7.6), 300 mM NaCl, 10% glycerol, and 0.5 mM EDTA with the addition of TEV protease. The resulting protein solution was loaded twice onto a Ni-NTA resin column pre-equilibrated with 50 mM HEPES (pH 7.6), 300 mM NaCl, and the flow-through fraction was collected. The purified proteins were concentrated with Amicon centrifugal filter devices (30 KDa MWCO, Millipore), and used for in vitro assays.

### General enzyme assays of wild type and mutants of *Pu*CGD with synthesized 3”-oxo-puerarin

For enzyme assays, 0.1 mM 3”-oxo-puerarin was incubated in 50 mM KPi buffer (pH 7.4) containing 10 μM purified recombinant *Pu*CGD or mutants. The enzyme reactions were performed at 37 °C for 30 min and then quenched with 50 μL of MeOH. The reaction mixtures were analyzed by HPLC. All measurements were conducted in triplicate.

### General enzyme assays of *Pu*CGD, *Eu*CGD, *Mi*CGD*, Ag*CGD1, *Ag*CGD2, and *Mt*CGD with oxidoreductases

For enzyme assays of *Pu*CGD and *Eu*CGD, 0.1 mM substrate and 0.2 mM 3-oxo-glucose were incubated with 10 μM DgpA or DfgE and *Pu*CGD, *Eu*CGD, or variants in 50 mM KPi buffer (pH 7.4). The enzyme reactions were performed at 37 °C for 30 min and then quenched with 50 μL of MeOH. The reaction mixtures were analyzed by HPLC. All measurements were conducted in triplicate. For enzyme assaying of *Mi*CGD*, Ag*CGD1, *Ag*CGD2, and *Mt*CGD, the 100 μl reaction mixture consisted of each of these enzymes, 10 mM Tris-HCl (pH 8.0), and 0.1 mM substrate, and was incubated at 28 °C. The reaction was stopped by adding an equal volume of acetonitrile. The amounts of reaction products were determined by HPLC-PDA or LC-MS.

### HPLC and LC/MS analyses

A sample was applied to a Cosmosil πNAP column (4.6 × 150 mm; Nacalai Tesque Co., Inc., Kyoto, Japan). HPLC and LC/MS analyses were performed using a Prominence system with a photodiode array detector (SPD-M20A) and an LCMS-8040 (Shimadzu, Kyoto, Japan). The HPLC conditions were as follows: flow rate, 1 ml min^−1^; solvent A, 0.1% (v/v) HCOOH; and solvent B, methanol. After column equilibration with 50% solvent B, a linear gradient system of solvent B (50% to 100%) was applied over 13 min, followed by 100% solvent B for 2 min.

### Temperature- and pH-optimization assays

To determine the optimal assay parameters for *Pu*CGD, 0.1 mM 3”-oxo-puerarin was incubated in 50 mM KPi buffer (pH 7.4) containing 10 μM purified recombinant *Pu*CGD. For the *Eu*CGD reaction, 0.1 mM homoorientin and 0.2 mM 3-oxo-glucose were incubated with 20 μM DfgE in 50 mM KPi buffer (pH 7.4) for 2.5 h, and then 20 μl of a 3”-oxo-homoorientin-containing solution was used as the substrate of *Eu*CGD. The reactions were performed at 25, 30, 37, 42, 50, 60, and 70 °C for 1 h, and then quenched with 50 μL of MeOH. The reaction mixtures were analyzed by HPLC. For thermal dependency estimation of *Mi*CGD*, Ag*CGD1, *Ag*CGD2, and *Mt*CGD, the 100 μl reaction mixture consisted of 1 μl of 1.1 mg ml^−1^ *Mi*CGD (or 1.9 mg ml^−1^ *Ag*CGD1, 0.38 mg ml^−1^ *Ag*CGD2, or 0.063 mg ml^−1^ *Mt*CGD), 1 μl of 1 M Tris-HCl (pH 8.0), and 1 μl of 10 mM carminic acid (or homoorientin for *Ag*CGD1 and *Mt*CGD, and orientin for *Ag*CGD2). The experiments were performed in triplicate at 20, 25, 30, 35, 40, 45, 50, 60, and 70 °C for 60 min (or 15 min for *Ag*CGD1 and *Mt*CGD, and 5 min for *Ag*CGD*2*). A Chill Heat CHT-101 (IWAKI Asahi Techno Glass, Tokyo, Japan) was used for the incubations from 10 to 50 °C, and a Dry Thermo Unit DTU-1B (TAITEC, Tokyo, Japan) was used for the incubations at 60 and 70 °C. The amounts of reaction products were determined by HPLC-PDA.

To optimize the reaction pH of *Pu*CGD and *Eu*CGD, the enzymatic reactions were carried out in various reaction buffers with pH values ranging from 4.0–6.0 (citric acid-sodium citrate buffer), 6.0–8.5 (K_2_HPO_4_-KH_2_PO_4_ buffer), 7.0–9.0 (Tris-HCl buffer), and 9.0–11.0 (CAPSO). All measurements were conducted in triplicate. For pH dependency estimations, the 100 μl reaction mixture consisted of 1 μl of 1.1 mg ml^−1^ *Mi*CGD (or 3.8 mg ml^−1^ *Ag*CGD1, 3.8 mg ml^−1^ *Ag*CGD2, or 3.2 mg ml^−1^ *Mt*CGD), 25 μl of 0.4 M Britton-Robinson buffer [pH 6-8 (0.5 pH units)], and 2 μl of 10 mM carminic acid (or homoorientin for *Ag*CGD1 and *Mt*CGD, and orientin for *Ag*CGD). The experiments were performed for 60 min (or 120 min for *Ag*CGD1 and 15 min for *Ag*CGD2) at 28 °C. The reaction was stopped by adding 100 μl of acetonitrile. The amounts of reaction products were determined by HPLC-PDA.

### Metal ion dependence experiment

The purified recombinant *Pu*CGD, *Mi*CGD*, Ag*CGD1, and *Ag*CGD2 complex was treated with 5 mM EDTA and 5 mM Tiron at 4 °C with slow rotation for 3 days, and then the excess EDTA was removed by dialysis with 20 mM HEPES (pH 7.6) buffer containing 100 mM NaCl overnight. For *Eu*CGD, the enzyme was incubated in buffer comprising 20 mM HEPES (pH 7.6) buffer, 100 mM NaCl, 20 mM EDTA, and 20 mM Tiron for 3 days at 4 °C. The enzyme reactions were performed without or with 1 mM divalent metal ion (Mn^2+^, Ni^2+^, Mg^2+^, Fe^2+^, Cu^2+^, Co^2+^, Zn^2+^, and Ca^2+^). All measurements were conducted in triplicate. The reactions were terminated with MeOH and the mixtures were centrifuged at 20,000 × g for 10 min for further HPLC analysis.

### Substrate specificity

Each of the following compounds was examined for substrate specificity analysis at a final concentration of 0.1 mM: carminic acid, mangiferin, homoorientin, isovitexin, puerarin, and orientin. Each of these compounds was added to the standard assay mixture. The production of the reaction products was detected by LC/MS. The reaction products of carminic acid were determined by mass spectrometry and NMR spectroscopy. Other reaction products were identified by mass spectrometry. For kinetic analysis of *Pu*CGD and *Eu*CGD, enzymatically prepared substrates (0.63, 1.3, 2.5, 5, 10, and 20 mM for **1**, **3**, and **4**, and 0.16, 0.32, 0.63, 1.3, 2.5, and 5 mM for **2** and **5**) were incubated with 2 μM of each enzyme for 2 min at 30 °C (only for **2**, 5 μM of *Pu*CGD was incubated for 90 min due to the low activity). And for kinetic analysis of *Mi*CGD, *Ag*CGD1, *Ag*CGD2, and *Mt*CGD, enzymatically prepared substrates (0.25, 0.5, 1, 2, 5, 10, and 20 mM) were incubated with 2 μM of each enzyme for 30 min at 30 °C. Each reaction was performed in triplicate. GraphPad Prism 9 (GraphPad Prism Software Inc., San Diego, CA) or Sigma Plot 12.5 (Systat Software Inc., San Jose, CA) was used for statistical data analysis.

### Draft genome sequence of a carminic acid-metabolizing microorganism

Total DNA from *Microbacterium* sp. 5-2b, which was isolated by an enrichment method using carminic acid as a sole carbon source, was prepared as follows. The strain was cultured at 28 °C for 48 h in 100 ml of 1/10 × 2YT media [0.1% (w/v) Bacto Yeast Extract (DIFCO), 0.16% (w/v) Bacto Tryptone (DIFCO), and 0.05% (w/v) NaCl]. Cells were harvested by centrifugation, washed with 10 mM Tris-HCl buffer (pH 8.0) containing 1 mM EDTA and 100 mM NaCl, and then suspended in 10 ml of 50 mM Tris-HCl buffer (pH 8.0) containing 10 mM EDTA and 15% (w/v) sucrose. The suspension was incubated with 7 mg ml^−1^ of lysozyme at 37 °C for 3 h, and then 2 ml of 0.5 M EDTA (pH 8.0), 2 ml of 10% SDS and 2.7 mg of proteinase K were added to the solution, which was incubated at 55 °C for 16 h. DNA was purified by extracting the lysate with phenol/chloroform/isoamyl alcohol (25/24/1; v/v/v), followed by precipitation with isopropanol, treatment with RNase, and then reprecipitation with ethanol. Draft genome sequencing of strain 5-2b was performed using an Illumina HiSeq platform (Hokkaido System Science Co., Ltd., Sapporo, Japan). We obtained 44.6 million reads of a 100 bp paired-end read. A total of 477 contigs comprising 189 ~1,692,717 bp were assembled. The draft genome sequence was annotated with MiGAP (http://www.migap.org).

### *Mi*CGD homologs

Using the protein sequence of *Mi*CGD^α^ (Supplementary Table 6) as a query, a BLAST search was performed against the database of the nucleotide collection. Among the protein sequences of the top 100 ORFs from the BLAST results, we chose ten microorganisms with genome data available online. Among them, the two *Mi*CGD^α^ homologs from *Arthrobacter globiformis* NBRC12137, and one *Mi*CGD^α^ homolog from *Microbacterium trichothecenolyticum* NBRC15077 were designated as *Ag*CGD1, *Ag*CGD2, and *Mt*CGD, respectively. They were cloned and heterologously expressed in *E. coli* BL21 Star (DE3).

### Size-exclusion chromatography-multiangle static light scattering

SEC-MALS analyses were performed with a WTC-030S5 (WYATT Technology) using a LaChrom Elite high-performance liquid chromatography system (Hitachi). Light scattering and the refraction index were measured using a Dawn Heleos II detector (Wyatt Technology) and a RI-101 detector (Shodex), respectively. The column was equilibrated at 20 °C with 20 mM Tris-HCl buffer, pH 8.0, containing 100 mM NaCl. Samples (2 mg ml^−1^) were injected at the buffer flow rate of 0.5 mL/min. The obtained data were recorded and processed using the ASTRA 6.1 software (Wyatt Technologies).

### Crystallization and structure determination

*Pu*CGD crystals were obtained at 20 °C in 24% (w/v) PEG4000, 100 mM Tris-HCl (pH 8.5) with 15 mg ml^−1^ of purified *Pu*CGD, by the sitting-drop vapor-diffusion method. *Eu*CGD crystals were obtained at 20 °C in 1190 mM (NH_4_)_2_SO_4_, 100 mM Tris-HCl (pH 8.5) with 15 mg ml^−1^ of purified *Pu*CGD, by the sitting-drop vapor-diffusion method. The crystallization conditions for *Ag*CGD2 were initially screened using Crystal Screen 1 and 2 (Hampton Research), Wizard Screens I and II (Rigaku), PEGsII (Qiagen), Index (Hampton Research), PEGIon/PEGIon2 (Hampton Research), and a Protein complex suite (Qiagen) with a Protein Crystallization System 2 (PXS2) at the Structural Biology Research Center, High Energy Accelerator Research Organization, Japan^39^. Diffraction quality crystals were obtained in 25–30% (w/v) PEG4000, 0.1 M MES (pH 6.5), and 0.2 M potassium iodide at 20 °C. The crystals of *Pu*CGD and *Eu*CGD were transferred into the cryoprotectant solution (reservoir solution containing 25% (v/v) glycerol). The crystals of *Ag*CGD2 were cryoprotected by immersion in a solution containing 15 % (w/v) PEG 1000, 21% (w/v) PEG4000, 60 mM MES (pH 6.5), and 60 mM potassium iodide.

The X-ray diffraction data sets of *Pu*CGD and *Eu*CGD were collected at −178 °C using a beam wavelength of 1.08 Å at BL-1A (Photon Factory, Tsukuba, Japan). The diffraction data sets were processed and scaled using the XDS program package and Aimless of ccp4^40,41^. X-ray diffraction data of *Ag*CGD2 for the native SAD method were collected at −178 °C, using an Eiger X16M detector on BL-17A of the Photon Factory, KEK (Tsukuba, Japan). The diffraction data were processed and scaled by XDS and XSCALE, respectively^41^. The phases were determined using the Crank2 program, by the native SAD method^42^. Crystallographic refinement and model building were performed using PHENIX.refine^43^ and Coot^44^. During the crystallographic refinement, a strong peak was found for the active site. A difference anomalous Fourier map that was calculated using diffraction data collected at (1.8900 Å) wavelength of manganese showed a strong peak at the active site. However, no peaks were found in the difference anomalous Fourier map calculated from the low remote (1.9000 Å) wavelength data.

Crystals of the homoorientin complex were prepared by soaking crystals of *Ag*CGD2 in a crystallization solution containing 10 mM homoorientin, for 2 h. Crystals of the homoorientin complex were cryoprotected with a solution containing 10 mM homoorientin, 15 % (w/v) PEG 1000, 21% (w/v) PEG4000, 60 mM MES (pH 6.5), and 60 mM potassium iodide, for 15 s. Diffraction data sets of *Ag*CGD2 were collected at -178 °C using a Pilatus 2M-F detector on NE3A of the PF-AR, KEK (Tsukuba, Japan). Data processing and scaling were performed as described above.

The initial phase of the *Pu*CGD structure was determined by molecular replacement, using the cryo-EM structure of *Pu*CGD as the search model. Molecular replacement was conducted with Phaser in PHENIX^45^. The initial phase was further calculated with AutoBuild in PHENIX^46^. The crystal structure of the *Ag*CGD2-homoorientin complex was determined by the molecular replacement method, using Molrep^47^. Crystallographic refinement was performed using PHENIX.refine^43^ and Coot^44^. The final crystal data and intensity statistics are summarized in Supplementary Table 3. The Ramachandran statistics are as follows: 96.5% favored, 3.5% allowed for *Pu*CGD and 96.8% favored, 3.2% allowed for *Eu*CGD, 97.3% favored, 2.7% allowed for *Ag*CGD2 with homoorientin. All crystallographic figures were prepared with PyMOL (DeLano Scientific, http://www.pymol.org).

The three-dimensional model structure of orientin was generated with the CHEM3D ULTRA 10 program (Cambridge Soft), and the geometries were optimized with the elbow tool in phenix^48^. Orientin or homoorientin was manually added to the active site to fit the aglycone part of homoorientin, with Coot^44^. The parameters of orientin for the refinement were obtained with the PRODRG server (http://davapc1.bioch.dundee.ac.uk/prodrg/).

### Cryo-EM sample preparation and data collection

The purification of *Pu*CGD and *Eu*CGD for cryo-EM analysis was performed in the same manner as for crystallization. The concentrations of *Pu*CGD and *Eu*CGD were adjusted to 37.2 μM and 44.8 μM (concentration of heterooctomer). For cryo-grid preparation, 3 μl samples were applied onto a holey carbon grid (Quantifoil, Cu, R1.2/1.3, 300 mesh), which was rendered hydrophilic by a 30 sec glow-discharge in air (11 mA current) with a PIB-10 plasma ion bombarder (Vacuum Device Inc., Ibaraki, Japan). The grid was blotted for 5 sec at force 20 for *Pu*CGD and 20 sec at force 0 for *Eu*CGD in 100% humidity at 18 °C, and then plunged into liquid ethane using a Vitrobot Mark IV (Thermo Fisher Scientific). The cryo-EM data collection was conducted using a Talos Arctica microscope (Thermo Fisher Scientific) at 200 kV in the nanoprobe mode, using the EPU software at the cryo-EM facility in KEK (Ibaraki, Japan). For *Pu*CGD, the movies were collected by a 4k x 4k Falcon 3EC direct electron detector (electron counting mode) at a nominal magnification of 120,000× (0.88 Å/pixel), with an accumulated dose of 50 electrons per Å^2^ over 49 frames. For *Eu*CGD, the movies were collected by the Falcon 3EC direct electron detector at a nominal magnification of 150,000× (0.69 Å/pixel) magnification, with an accumulated dose of 50 electrons per Å^2^ over 62 frames.

### Cryo-EM data processing software

For the cryo-EM analysis, motion correction and dose-weighting were performed using the MotionCor2 frame alignment program^49^. The contrast transfer function (CTF) parameters were estimated using Gctf^50^ and CTFFIND4^51^. The particles were picked using SPHIRE-crYOLO with the general model^52^. The ctflimit function^53^ implemented in a Python module, morphology.py, of SPARX/SPHIRE^54,55^ was used to calculate the smallest box size that ensures no CTF aliasing in the reciprocal space, up to the target resolution for a given defocus value. RELION3^56^ and RELION 3.1.0-beta were used for all other SPA steps: reference-free 2D classification, ab initio reconstruction, 3D classification, 3D refinement, CTF refinement for the refinement of higher-order aberrations, anisotropic magnification, per-particle defocus and beam tilt, and Bayesian polishing for beam-induced motion corrections^57^. After each of the 3D refinements, the “gold-standard” FSC resolution with the 0.143 criterion^58^ in RELION was used as a global resolution estimation with phase randomization, to account for possible artifactual resolution enhancement caused by solvent mask^59,60^. The local resolutions of the 3D cryo-EM maps were estimated using RELION’s own implementation. UCSF Chimera^61^ and e2display.py of EMAN2^62^ were used for the visualization of the output 2D/3D images. The detailed methods for cryo-EM processing are written in *Supplementary Methods*.

### Mutagenesis of *Pu*CGD and *Mi*CGD

The plasmids expressing the mutant enzymes of *Pu*CGD and *Mi*CGD were constructed with a QuikChange Site-Directed Mutagenesis Kit (Stratagene) and a KOD-plus mutagenesis kit (Toyobo Co., Ltd., Osaka, Japan), respectively, according to the manufacturer’s protocol. The variants of *Pu*CGD and *Mi*CGD were transformed into *E. coli* BL21(DE3) and BL21-Star (DE3), respectively. The expression, purification, and enzyme reactions of all variants were performed in the same manners as for the wild-type enzymes. The primers used for the construction of mutants are listed in Supplementary Table 5.

### Preparation of manual docking model structures

The three-dimensional model structures of orientin and homoorientin were generated by the CHEM3D ULTRA 10 program (Cambridge Soft), and their geometries were optimized with the elbow tool in phenix. Orientin or homoorientin was manually added in the active site to fit the position of the C-C bond between *C*-glycoside and aglycone. Then, the conformation of ligands was manually modified to avoid the close contacts between the ligand and the active site residues. Here, the positional relationship among the C-C bond, His149, and Glu307 (numbering of *Ag*CGD2), was defined to be almost the same in all models. It is noted that we did not modify the conformation of the active site residues.

### Reporting summary

Further information on experimental design is available in the Nature Research Reporting Summary linked to this paper.

## Supplementary information

Supplementary Information

Reporting summary

## Data Availability

Source data are provided with this paper. The data generated in this study are provided in the Supplementary Information/Source Data file. The crystallographic data that support the findings of this study are available from the Protein Data Bank (http://www.rcsb.org). The coordinates and the structure factor amplitudes for the apo structures of *Pu*CGD, *Eu*CGD, *Ag*CGD2, and *Ag*CGD2 complexed with homoorientin were deposited under accession code 7EXZ^63^, 7EXB^64^, 7DNM^65^, and 7DNN^66^, respectively. The cryo-EM maps and the atomic coordinates for *Pu*CGD and *Eu*CGD determined by cryo-EM have been deposited in Electron Microscopy Data Bank (EMDB, https://www.ebi.ac.uk/pdbe/emdb/) and PDB with accession codes EMD-30808 and EMD-30809, and 7DRD^67^ and 7DRE^68^, respectively. Source data are provided with this paper.

## Source data

Source Data
