# Supplementary material for: C-Glycoside metabolism in the gut and in nature: Identification, characterization, structural analyses and distribution of C-C bond-cleaving enzymes
Source: Nat Commun. 2021 Nov 2;12:6294. doi: 10.1038/s41467-021-26585-1 (PMC8563793; doi:10.1038/s41467-021-26585-1)
Supplement: Supplementary file 1 — Supplementary Information [file 41467_2021_26585_MOESM1_ESM.pdf]

Supplementary information

C-Glycoside metabolism in the gut and in nature: Identification, characterization, structural analyses and distribution of C-C bond-cleaving enzymes

Takahiro Mori[1,2,3,#], Takuto Kumano[4,5,#], Haibing He[1,#], Satomi Watanabe[4,#], Miki Senda[6], Toshio Moriya[6], Naruhiko Adachi[6], Sanae Hori[4], Yuzu Terashita[4], Masato Kawasaki[6], Yoshiteru Hashimoto[4,5], Takayoshi Awakawa[1,2], Toshiya Senda[6,7,*], Ikuro Abe[1,2,*], Michihiko Kobayashi[4,5,*]

[1] Graduate School of Pharmaceutical Sciences, The University of Tokyo, 7-3-1 Hongo, Bunkyo-ku, Tokyo 113-0033, Japan

[2] Collaborative Research Institute for Innovative Microbiology, The University of Tokyo, Yayoi 1-1-1, Bunkyo-ku, Tokyo 113-8657, Japan

[3] PRESTO, Japan Science and Technology Agency, Kawaguchi, Saitama 332-0012, Japan

[4] Graduate School of Life and Environmental Sciences, University of Tsukuba, 1-1-1 Tennodai, Tsukuba, Ibaraki 305-8572, Japan

[5] Microbiology Research Center for Sustainability, University of Tsukuba, 1-1-1 Tennodai, Tsukuba, Ibaraki 305-8572, Japan

[6] Structural Biology Research Center, Institute of Materials Structure Science, High Energy Accelerator Research Organization (KEK), 1-1 Oho, Tsukuba, Ibaraki 305-0801, Japan

[7] Faculty of Pure and Applied Sciences, University of Tsukuba, 1-1-1 Tennodai, Ibaraki 305-8571, Japan

# These authors contributed equally to this work.

* Corresponding authors

Supplementary Tables

Supplementary Table 1. Sequence similarity of *Pu*CGD homolog enzymes.

A. Homolog enzymes of α-subunit of *Pu*CGD

Organism	Accession No. (Enzyme name)	Length (aa)	Identity (%)	Similarity (%)	E-value
PUE strain	BBG22495.1 (DgpC; α-subunit of *Pu*CGD)	324	100	100	0
Eubacterium cellulosolvens	WP_004604489.1 (DfgA; α-subunit of *Eu*CGD)	290	34	50	$3e^{-38}$
Klebsiella pneumoniae	WP_023290705.1	337	35	53	$9e^{-22}$
Enterococcus durans BDGP3	WP_081133590.1	323	48	70	$7e^{-117}$
Microbacterium sp. 5-2b	LC387599 (CarB; α-subunit of *Mi*CGD)	348	29	49	$2e^{-41}$
Arthrobacter globiformis NBRC12137	WP_003803559.1 (*Ag*CarB1: α-subunit of *Ag*CGD1)	367	26	45	$5e^{-31}$
	WP_003803556.1 (*Ag*CarB2: α-subunit of *Ag*CGD2)	352	26	43	$8e^{-33}$
Microbacterium trichothecenolyticum NBRC15077	WP_003803556.1 (*Mt*CarB: α-subunit of *Mt*CGD)	352	27	45	$8e^{-33}$
Arthrobacter sp. Rue61a	WP_014921466.1	366	27	45	$4e^{-31}$
Microbacterium pygmaeum DSM23142	WP_091486435.1	369	27	44	$1e^{-31}$
Agromyces flavus CPCC202695	WP_092669325.1	357	27	43	$5e^{-32}$
Sphingobium yanoikuyae S72	WP_010338599.1	335	30	49	$3e^{-47}$
Leifsonia sp. 98AMF	WP_089877376.1	323	29	49	$8e^{-43}$

B. Homolog enzymes of β-subunit of *Pu*CGD

Organism	Accession No. (Enzyme name)	Length (aa)	Identity (%)	Similarity (%)	E-value
PUE strain	BBG22494.1 (DgpB; β-subunit of *Pu*CGD)	142	100	100	$6e^{-109}$
Eubacterium cellulosolvens	WP_004604488.1 (DfgB; β-subunit of *Eu*CGD)	147	38	56	$9e^{-23}$
Klebsiella pneumoniae	WP_023290704.1	130	25	44	$5e^{-38}$
Enterococcus durans BDGP3	WP_081133591.1	143	43	63	$2e^{-45}$
Microbacterium sp. 5-2b	LC387600 (CarC; β-subunit of *Mi*CGD)	132	30	52	$2e^{-15}$
Arthrobacter globiformis NBRC12137	WP_003803560.1 (*Ag*CarC1: β-subunit of *Ag*CGD1)	131	27	50	$4e^{-10}$
	WP_003803554.1 (*Ag*CarC2: β-subunit of *Ag*CGD2)	132	30	50	$2e^{-14}$
Microbacterium trichothecenolyticum NBRC15077	WP_045298609.1 (*Mt*CarC: β-subunit of *Mt*CGD)	131	29	48	$2e^{-11}$
Arthrobacter sp. Rue61a	WP_014921465.1	131	27	50	$3e^{-10}$
Microbacterium pygmaeum DSM23142	WP_091486431.1	131	32	51	$4e^{-15}$
Agromyces flavus CPCC202695	WP_092669324.1	134	28	44	$1e^{-10}$
Sphingobium yanoikuyae S72	WP_069336345.1	130	34	54	$1e^{-24}$
Leifsonia sp. 98AMF	WP_089877379.1	138	34	54	$2e^{-16}$

Supplementary Table 2. SEC-MALS analyses of *Pu*CGD, *Eu*CGD, *Mi*CGD, *Ag*CGD1, *Ag*CGD2, and *Mt*CGD.

	Mw (calcd: α-subunit+β-subunit)	Mw (Obvious)	Solution state
*Pu*CGD (DgpB-C complex)	52,133	214,400 (± 0.52%)	heterooctomer
*Eu*CGD (DfgA-B complex)	54,432	216,600 (± 1.29%)	heterooctomer
*Mi*CGD (CarB-C complex)	55,130	49,030 (± 0.54%)	heterodimer
*Ag*CGD1 (*Ag*CarB1-C1 complex)	56,209	58,100 (± 2.05%)	heterodimer
*Ag*CGD2 (*Ag*CarB2-C2 complex)	56,663	54,170 (± 5.63%)	heterodimer
*Mt*CGD (*Mt*CarB1-C1 complex)	56,043	57,610 (± 2.34%)	heterodimer

Supplementary Table 3. Data collection and refinement statistics of X-ray crystallographic data.

	*Pu*CGD-apo (PDB 7EXZ)	*Eu*CGD -apo (PDB 7EXB)	*Ag*CGD2 apo (PDB 7DNM)	*Ag*CGD2 with homoorientin (PDB 7DNN)
**Data collection**				
Space group	$P2_12_12_1$	$P6_422$	$P2_12_12$	$P2_12_12$
Cell dimensions				
a, *b*, *c* (Å)	86.5, 156.7, 158.9	139.0, 139.0, 227.0	71.7, 100.6, 136.3	73.9, 102.5, 136.1
α, β, γ (°)	90, 90, 90	90, 90, 120	90, 90, 90	90, 90, 90
Resolution (Å)	46.9-2.50 (2.55-2.50)*	47.1-2.40 (2.47-2.40)*	47.2-2.30 (2.42-2.30)*	47.97-2.25 (2.37-2.25)*
R_{merge} (%)	12.2 (81.5)	14.2 (144.9)	4.2 (26.5)	5.4 (85.2)
$<I / \sigma I>$	12.0 (2.1)	27.4 (2.8)	28.2 (7.7)	17.5 (2.0)
Completeness (%)	100.0 (100.0)	100.0 (99.9)	96.6 (91.6)	99.8 (99.9)
Multiplicity	6.9 (5.9)	36.9 (22.8)	6.8 (6.6)	4.2 (4.2)
**Refinement**				
Resolution (Å)	46.9-2.50	47.1-2.40	47.2-2.30	36.2-2.25
No. reflections	75,414	51,231	81,711	94,646
R_{work} / R_{free}	0.182/0.233	0.178/0.203	0.180/0.223	0.196/0.247
No. atoms				
Protein	14584	3,567	7,372	7,319
Ligand/ion	183	21	4	36
Water	475	329	178	173
B-factors (Å^2)				
Protein	41.6	41.6	40.6	55.8
Ligand/ion	47.3	57.6	35.1	46.2
Water	38.5	45.1	39.2	49.0
R.m.s. deviations				
Bond lengths (Å)	0.008	0.007	0.008	0.008
Bond angles (°)	0.948	0.899	0.996	0.935

*One crystal was used for collection. Values in parentheses are for highest-resolution shell.

Supplementary Table 4. Data collection and refinement statistics of cryo-EM data.

	*Pu*CGD (EMDB-30808) (PDB 7DRD)	*Eu*CGD (EMDB-30809) (PDB 7DRE)
**Data collection and processing**		
Microscope	Talos Arctica	Talos Arctica
Voltage (kV)	200	200
Detector	Falcon 3EC	Falcon 3EC
Magnification	120,000	150,000
Pixel size (Å) (calibrated)	0.88 (-)	0.69 (0.676)
Automation software	EPU	EPU
Total exposure (e–/Å^2)	50	50
Exposure rate (e–/Å^2 frame)	1.02	0.81
No. of frames	49	62
Defocus range (µm)	-1 to -2.5	-0.6 to -1.5
Symmetry imposed	D2	D2
No. of collected micrographs	2,122	1,664
No. of selected micrographs	1,963	1,614
No. of particles for 2D classification	857,817	330,866
No. of particles for 3D classification	597,534	232,605
No. of particles for 3D refinement	56,924	60,587
Map resolution (Å)	2.85	2.54
FSC threshold	0.143	0.143
**Refinement**		
Initial model used (PDB code)	none	none
Model composition		
Non-hydrogen atoms	12,709	12,604
Protein residues	1604	1572
Ligands	0	0
B factors (min/max/mean, Å^2)		
Protein	28.6/86.9/48.8	18.0/51.9/31.2
Ligand	0/0/0	0/0/0
R.m.s. deviations from ideal values		
Bond lengths (Å)	0.004	0.005
Bond angles (°)	0.598	0.554
Validation		
MolProbity score	1.24	1.36
Clashscore	4.64	4.6
Poor rotamers (%)	1.48	1.49
Ramachandran plot		
Favored (%)	98.2	98.7
Allowed (%)	1.8	1.3
Disallowed (%)	0	0

Supplementary Table 5. Primers used in this study.

**Primers used for cloning of *MiCGD, AgCGD1, AgCGD2* and *MtCGD*, and construction of mutants**	
CarB Fw	TAAGAAGGAGAGATATACATATGACCGCCCTCGCCCTGCCCCGCCTCG
CarB Rv	TTGTCGACGGAGCTCGAATTCTCATCGCACGCCCGCCTCGGCCTCG
CarC Fw	TAAGAAGGAGAGATATACATATGTTCAGAGAACGCATCATCGTTCAGGACTCG
CarC Rv	TTGTCGACGGAGCTCGAATTCTCATGCGGTGAGCTCCTTGACGTCCATG
*MiCGD*_Fw	TAAGAAGGAGAGATATACATATGTTCAGAGAACGCATCATCGTTCAGGACTCG
*MiCGD*_Rv	GTGGTGGTGGTGGTGCTCGAGTCGCACGCCCGCCTCGGCTCGA
*AgCGD*1_Fw	TAAGAAGGAGATATACATATGATCGCCGACCGCATAATCGAGCAC
*AgCGD*1_Rv	CTCGAGTGCGGCCGCAAGCTTGCCGCGGACGGGCTGGGCGA
*AgCGD*2_Fw	TAAGAAGGAGATATACATATGTCAGAAGGCATCGCCGGCTCG
*AgCGD*2_Rv	CTCGAGTGCGGCCGCAAGCTTTGCGAGAACGACCTTGCGCTCG
*MtCGD*_Fw	TAAGAAGGAGATATACATATGATCCCCGATCGCATCATCGAGCAGG
*MtCGD*_Rv	CTCGAGTGCGGCCGCAAGCTTTGCGTGTGCTCCTTCGGTGTTGG
MiCGD$^{\alpha}$ E145A-F	ATGGGCATGGcGATCCACAGC
MiCGD$^{\alpha}$ E145A-R	GGTCAGATCGAGCTTCTC
MiCGD$^{\alpha}$ H147A-F	CATGGAGATCgcCAGCCCGCAC
MiCGD$^{\alpha}$ H147A-R	CCCATGGTCAGATCGAGC
MiCGD$^{\alpha}$ D177A-F	TTCATCCCGGcCTGGGGCGCC
MiCGD$^{\alpha}$ D177A-R	TCCGAGATGCGGGGATCCG
MiCGD$^{\alpha}$ H275A-F	GTGCACGTGgcCGGCAAGTTC
MiCGD$^{\alpha}$ H275A-R	GACCCAGGGCATGATCGC
MiCGD$^{\alpha}$ E311A-F	ATTTCCAGTGcGTGGGAGGGCTGG
MiCGD$^{\alpha}$ E311A-R	GTAGCCGGTGTAGCCCGC
MiCGD$^{\alpha}$ E313A-F	AGTGAGTGGGcGGGCTGGCAC
MiCGD$^{\alpha}$ E313A-R	GGAAATGTAGCCGGTGTAGC
**Primers used for cloning of *dgpA, PuCGD, EuCGD* and *dfgE,* and construction of mutants**	
DgpA-F	AAGGAGATATACATATGTCCAAGCTGAAAATTG
DgpA-R	CTCGAGTGCGGCCGCAAGCTTTCAGAACTTAATG
PuCGD$^{\beta}$-F	GAAGGAGATATACATATGGGCTTAGCGTTACG
PuCGD$^{\beta}$ -R	CTTTTTATCTCCTCCCTAATTCTGCACATTC
PuCGD$^{\alpha}$-F	AGGGAGGAGATAAAAAGATGTCTAACGTGAAAC
PuCGD$^{\alpha}$-R	GTGCGGCCGCAAGCTTGTCCACAAAGTTGTG
EuCGD$^{\alpha}$-F	GAAGGAGATATACATATGTATCGTTATGAGAAG
EuCGD$^{\alpha}$-R	TTATCTCCTCCCTAGTCTCCTAATAAGTTC
EuCGD$^{\beta}$-F	CTAGGGAGGAGATAAAAAGATGGAGAAACAGG
EuCGD$^{\beta}$-R	GTGCGGCCGCAAGCTTCACGATCAACAG
DfgE-F	GAAGGAGATATACATATGGCGCAGGACATTCG
DfgE-R	GTGCGGCCGCAAGCTTAAGCTTCTCGAAGTC
PuCGD$^{\alpha}$ E141A-F	CGTCAAAGTTGGTATTgccGTGCATAATCCG
PuCGD$^{\alpha}$ E141A-R	GGATTATGCACggcAATACCAACTTTGACG
PuCGD$^{\alpha}$ H143A-F	GTTGGTATTGAGGTGgccAATCCGGAAACC
PuCGD$^{\alpha}$ H143A-R	GGTTTCCGGATTggcCACCTCAATACCAAC
PuCGD$^{\alpha}$ D173A-F	GGTTGATCCCCgccTTTGGCTGTTTTGCCAAC
PuCGD$^{\alpha}$ D173A-R	GTTGGCAAAACAGCCAAAggcGGGGATCAACC
PuCGD$^{\alpha}$ H263A-F	CCATATTGCATCCACATGgcgGGCAAATATCACTACATG
PuCGD$^{\alpha}$ H263A-R	GTAGTGATATTTGCCcgcCATGTGGATGCAATATGGAATC
PuCGD$^{\alpha}$ K265A-F	GCATCCACATGCATGGCgccTATCACTACATGTACG
PuCGD$^{\alpha}$ K265A-R	CGTACATGTAGTGATAggcGCCATGCATGTGGATGC
PuCGD$^{\alpha}$ E301A-F	CGTGAGCGAATACgcgGAATACAACTCAGG
PuCGD$^{\alpha}$ E301A-R	GAGTTGTATTCcgcGTATTCGCTCACGATATAGC

The underlined letters represent the restriction sites.

Supplementary Table 6. Sequences of enzymes.

**Nucleotide sequences**	
MiCGD$^{\alpha}$	1 ATGACCGCCCTCGCCCTGCCCCGCCTCGGCGTGACGCTGTACAGCTTCACGCCCTACTACCACGCGCGTGAGTAC<br>76 TCCTTCGAGGACCTCATCCGCATCGCCGGAGAGCGCGACCTCGGCCCCGGCCTCGAGATCGTCGGCTACCAGAGC<br>151 ATCAAGGGCTTCCCGAAGCTACCTGACGGCTTCGTGAAGGACTTCCGCCGCCGGGTCGACGAGGCCGGTCTCGAA<br>226 CTGAGCGCGATGGGCGCCAACGCCGACGCGGGCATCCCGCACGACCGGCTGCTGAACGAGGACGAGCTCACCGAG<br>301 TACATGGCCCACCAGCTGCACACCGCCAAGGAGCTCGGCTTCCCGATCGTCCGCGTGCAGCACTCGGTCACGCCC<br>376 GACCTCATGGAGCGCCTGCTCCCCCTCGCGGAGAAGCTCGATCTGACCATGGGCATGGAGATCCACAGCCCGCAC<br>451 AGCGTGCACCACCCGAAGATCCAGGCTCTCGTCGAGCGCTACGAGAAGCTCGGATCCCCGCATCTCGGATTCATC<br>526 CCGGACTGGGGCGCCAGCCTCACCCGCCTGCCCCCGAGCGCGCTGCAGACCTATGCCGCGGCCGACGTACCGCGG<br>601 GAGCTGCTCGACGCCTACGACCGGCAGTGGGACGTGTTCCACGCCGAGGGCGTCATCACCACCGACGCCGAGCAG<br>676 GGCGCCCAGTTCCGTCGCATGCGCGAGCTCAACGAGCGGTTCGGGGGTGACGACGTCTCGGTGCGCATCGGCACG<br>751 AACGCGGTGGGCCTGTTCGGCCACCAGCGGCCAGAGGACTGGTCGGCGATCATGCCCTGGGTCGTGCACGTGCAC<br>826 GGCAAGTTCTACGGCATCGACGAGAACGGCGAGGAGCCCTCCGTGCCGCACGGCCTGCTGCTGCGCCAGCTCGTG<br>901 GACGCGGGCTACACCGGCTACATTTCCAGTGAGTGGGAGGGCTGGCACTGGAACACGACCGACGACCCGTTCGAG<br>976 ATGGTCGCCTGGCAGCACCGGCTCATGCGCAGGATCTTCGGTGAGATCGAGGCCGAGGCGGGCGTGCGATGA
MiCGD$^{\beta}$	1 GTGTTCAGAGAACGCATCATCGTTCAGGACTCGCTCGTCGCCGACGAGTCGGGATCCGCCTTCCGGATCCGCCTG<br>76 CCCTGGTACCGCGCGCTGCCGCTCAGCACGATCGAGGAGCTCTCGGTCACGGTCGACGGCACGGCCTTCGACCCT<br>151 GCACGGCTACGGATCGCGGTGAACGACGGCGAGTGGGCCCTGGCCGAGGCGCAGCTGCGCACGGACGACGTCTGG<br>226 TTCGTCCTGGACGATGCGACCGTGCGGCTGCCCGGCCTCGTGCTCGACGCCGGTGCGCACGAAGTGCAGGCCACG<br>301 CTCTCGATGCGCATCCCCTATCTGCCGGTCGCCGGCAAGCCGCTCTCGATGGCCGAGACCGACCAGAAGCGCATG<br>376 GACGTCAAGGAGCTCACCGCATGA
**Protein sequences**	
*Mi*CGD$^{\alpha}$	1 MTALALPRLGVTLYSFTPYYHAREYSFEDLIRIAGERDLGPGLEIVGYQSIKGFPKLPDGFVKDFRRRVDEAGLE<br>76 LSAMGANADAGIPHDRLLNEDELTEYMAHQLHTAKELGFPIVRVQHSVTPDLMERLLPLAEKLDLTMGMEIHSPH<br>151 SVHHPKIQALVERYEKLGSPHLGFIPDWGASLTRLPPSALQTYAAADVPRELLDAYDRQWDVFHAEGVITTDAEQ<br>226 GAQFRRMRELNERFGGDDVSVRIGTNAVGLFGHQRPEDWSAIMPWVVHVHGKFYGIDENGEEPSVPHGLLLRQLV<br>301 DAGYTGYISSEWEGWHWNTTDDPFEMVAWQHRLMRRIFGEIEAEAGVR
*Mi*CGD$^{\beta}$	1 VFRERIIVQDSLVADESGSAFRIRLPWYRALPLSTIEELSVTVDGTAFDPARLRIAVNDGEWALAEAQLRTDDVW<br>76 FVLDDATVRLPGLVLDAGAHEVQATLSMRIPYLPVAGKPLSMAETDQKRMDVKELTA

**codon optimized nucleotide sequences for expression in *E. coli***	
PuCGD$^{\alpha}$	ATGTCTAACGTGAAACTTGGCGTAACCCTGTATAGCTTCAGTACGGAGTATTGTCAGGGTAAAAT<br>GACCTTAGAGGATTGTATTCGGACAGCGAAAGAACTGGGTGCAGCTGGTTTCGAGATTGTCGCC<br>ACACAGATGATTCCGTCCTATCCGTATGTGTCGGACAAATTCCTGGGTGAGCTGAAAAGCATTTG<br>CCAGTACTATGACATGGAACCTGTTTGCTATGGGGCTAACTGTGATCGCGGTCTGCGTGGCGATC<br>GCAATCTGACGGGTGATGAAATGGTCGCAATGGCGGTACGCGATATCAAGAATGCGCATAAAAT<br>GGGCTGCAAGGTTGTTCGCGAACAGTGGCTCATGGGTCCGGAAAACTTTGCCAAATTAGCGCCT<br>TTTGCCGAACACTATGGCGTCAAAGTTGGTATTGAGGTGCATAATCCGGAAACCCCGATTACGCA<br>ATCCACCAAAGACTACATTGCCGCTATCGATAAAACTGGCAGTAAATACCTTGGGTTGATCCCCGA<br>TTTTGGCTGTTTTGCCAACAAACCGAACAAGATGAATTGGGATAATGCGCTCGCAGATGGAGCG<br>GATAAGAAACTGCTGGAAATGGCCCGTGATATGAAATACGATAATGTACCCTATGACGAAGCGGT<br>TAAACGCCTGACTGCAGCAGGGGCGAAGAAAGTCGAATTGACGACCATGCGCGATATGTATACC<br>TTTCTGACCTTCAAGAAAGACGTGTCTGCTGAGCTCCAAGGACTGAAAGATATGATTCCATATTG<br>CATCCACATGCATGGCAAATATCACTACATGTACGAAAACTTACAGGAAGCGGCCATTCCATACGA<br>TGACATCATGAAAATCGTGTCAGAATCGGACTATGACGGCTATATCGTGAGCGAATACGAGGAAT<br>ACAACTCAGGCCATAGCATTGAAATGTTGCGTCGTCATCTGAAAATGATGCACAACTTTGTGGAC
PuCGD$^{\beta}$	ATGGGCTTAGCGTTACGCCTGAACTTTGTGGATGTCGTTTGCGATGACAGCTTGAAGAACTTTTG<br>GGCGAATGGGAAGAAAATCGGCTATCAGTTCGATGTTCGTCTGAGTTACTATCGCGGTCATTTCC<br>TGTCGACCATTGACGAAATTGGCGTCAAAGTGGATGGTGTGGATGTACCGGCAGAGAACATTTC<br>GCTGTGTCTTGATGGGAAAGAGTATGGCGTAGCTGAACTGCATGATCTGGTTAACGTCTTTTGGC<br>CGATTATCGAACCAGCCACTATCAAAGTGTTCCAACCTGGTGGTTTGAGCGAAGAAGAACACGA<br>TGTTGACTTTACGCTGTACTTTCGTAGTCCGTACATGGCCCTCTCAGAAACCGAGTATCAGTCCAT<br>TGACTCTTGCGGAAGCAAACGCCTGAATGTGCAGAAT
dgpA	ATGTCCAAGCTGAAAATTGGTATTATCGGTTGCGGAGGCATTGCTAACCAGAAACACTTCCCTGC<br>GTTAAAGAACAATGCTGATCTGAATGAGATTGTAGCCTTTTGTGACATCCAGATCGACCGCGCGG<br>AGAAGGCTGCTGCGGAGTTTGGTGCAGAGGGAGCTCAGGTGACAGCTGACTATAAAGAATTGC<br>TCGCGAACCCGGAAGTCGAAGTTGTACACGTCTGCACACCAAACGTTTCCCACTCTGAAATTACC<br>ATCGCCGCTTTTGAAGCTGGGAAACACGTATACTGTGAGAAGCCCATGAGTCACTCAACAGAGG<br>AAGCCGAGAAGATGGTTGAGGCCTGGAAGAAATCCGGTAAACAATTTACAATTGGTTACCAAAA<br>CCGTTTTCGTGAGGAAGTCATGAACCTCAAGAAAAGCTGCGATAAAGGGGAACTCGGTGAGAT<br>CTACTACGGTAAAGCGCATGCAGTACGTCGTCGTGCGGTTCCGACTTGGGGTGTGTTTATGGACA<br>AGGAGGCTCAGGGAGGCGGACCGCTCATCGACATTGGCACGCATGCCTTGGATATCACCTTATG<br>GTGCATGAACAACTACGATGTTGACAGCGTGACGGGCTCTGTGTTCTATAAACTGGGACAGAAA<br>GAGAACGGACCGGAAGGAAACTTGTTCGGCCCGTGGGACCCGAAAACATTTGAAGTGGAGGA<br>TAGTGCTGTGGGATTCGTGAAAATGAAGAATGGAGCTACTATTGGCCTTGAAGCGTCCTGGGCA<br>ATCAACATGCTCGATTCGCGCGAAGCATCTACGACTCTGTGTGGGACCGAGGCTGGGGCAGAGA<br>TTCATAGTGGCATGTCATACCCAAAGAACGAGTTGATCTACAATCGCGCACGTAATAACCAGCTTA<br>TGGAAGAAACCCTCAGTTCGGTGGGTTCGATTGCTTACTTTGCGGGAGGAGCGGGCGAAGAAG<br>GTACGGTTGACAATCGCCAGTGGTTGGAGGCGATCCAAAACGGGACTGAGCCCCTTGTGAAAC<br>CCGAAGAGGCCCTGGCAGTTACAAAGATCTTAGACGCCATCTATAAAAGCGCAAAGACGAATGA<br>GACCATTAAGTTC
EuCGD$^{\alpha}$	ATGTATCGTTATGAGAAGAAGGGCCCTAAGCGCGGAGTTGCGTTATACTCGTACTCAGCCGAATT<br>TGGGTTAACTAAGACGTTGGAAGATTGTTTCGAGGATCTTCACGATATGGGTGCTCACGGTATCG<br>AGATTTTAGCAAACACTCACATTGAAAACTATCCATACCCGACGGATGAATGGGTGGAGAAATGG<br>TGGCGCTTGTGTGACAAATACGAAATCGTTCCAGTGGAGTACGGAAACTGGATCGACTCTCATGT<br>ACTGGGAGATCGTGACCTGACTACCGAGGAGTCCGTTGAAATGCTCAAACGCGACATTCGCTTG<br>GCGCACCGTCTCGGTTTCACCGTCATGCGTACCAAGATGCCGGTCATTAATGACCTCTTAGAGCC<br>GGTAGAAAACTGGAAGGAAATCATCAAAGGTGCCCTGCCTCTCGCAGAAGAGTTAGGCATCAA<br>GATGTGTCCGGAGATCCATACGCCATCAAATTTGAAGGGAAAGCTGGTGAATGACTTCGTAGAG<br>TTTATCAAGGAGACAGGAACGAAGAACTTTGGTCTCAACATCGATTTTAGCGTCTTTCGCACCAG<br>TTTTGCTGAAGGTGAATGGGTGGACCCAAATTACACACCGAATAAGCCAGAAGACATTATTCCGC<br>TGCTGCCCTATGTGTATTGCTGTCATGCTAAATTTATCCACATGAGTGACGATTTTAAAGAAACCAC

	CATTCCCTACGAGGAGGTAGTCAAGACCATGGAAGATAACGGTTACGAGGGCTATTTGTTATCTG<br>AATACGAAGGCGCTGATAAATACGATGAGGGATACGAGGTAGGCCAGACACTCCGTAAACATCA<br>TATCTTACTCAAGAACTTATTAGGAGAC
EuCGD$^{\beta}$	ATGGAGAAACAGGTAATTCAGTCTGTAGGCTTCCGTAACATTAAGAACGGTAATGGCGAGATTAC<br>AGGCTTTCAGTTCAAGGTTAAACTTCCGTATTATCGCGGCGTCTTCTTATCCCAAATCCGCCCAGG<br>GACTTTGTTCGTCGACGGCCAGAAAATCGAGAAAGACCAAATTACCTGGACCATCAATGGCGAG<br>GAGTACACGAACCAGGAAATGCGTGGTGATTTCAAGACCCACTGGGCCACAACCAAACCCGCG<br>GTACTGAAAGTGAAAATGCCAGGTGGGTTAGCACAAGGGTATCATGACCTGAAATACGGATTTT<br>GTTTTACCAGCTCGTATATGCCGCCGATTATCCAAGATGGCCTGGACCCTGATAAAGAATCAATGG<br>TGTACATGCCAGAATTTGGGCATCACGTGAATGAACGCCGTCTGTTGATCGTG
dfgE	ATGGCGCAGGACATTCGCATCGCCATTGTGGGCCACGGTTTCATGGGACACGAGCATGAGAAAA<br>TGCTGTATGATTTTCCCGGTATTCGTCTGGTAGGTTTTAGCGATAAAGATCCTGCGCAATTAGAAG<br>ACGTTAAAGAGGGTCTTAAACGCTATCAATCGAATGAGGAGTTATTCGCCGATCCAGAAGTGGA<br>CGTCGTCATCATTGCGGCGAACAACAATCAGCATCGTGACTTGGTGATTCAGGCCGCGAATGCCG<br>GTAAGAATATCATTTGTGAAAAGCCGGTGGCGATGTCTCTGCAGGAGCTGGAAGAGATGGAAG<br>AGGCTGTAAAGCGCAATGGAGTAAAATTTACGGTACATCAACAGCGTCGCTTTGATCCTGACTTT<br>CGTACCGCGAAAGCCGTTTATGATTCCGGCACATTGGGTGAAGTGTATGCCATCAAGAACCAACT<br>GTACGGCTTCAATGGGAACATGCACGACTGGCACGTGTTTGTAGAAGAGGGTGGTGGAATGCTT<br>TATGACTGGGGTGTCCACCTGTTGGATCAGGTGTGTTTCATGTTCCCCGGTGCGAAGTTAAAGTC<br>AGTATTTGCGGACATTCGTAACGTGATCAATGATGAAGTCGATGACTACTTCAATATTATGTTACGC<br>TTCGACAATCAGGTGACAGCCACCGTCGAGCTGGGTACATACTACTTGGCGGATAAAGTGCAGG<br>ACAAATGGTTCGAACGCCACTGGTTCGTAGCTGGCAACAAGGGAACAGCTTACGTAGATGGGTT<br>CTTTCCTGAAGGCAAAATTGTACGTACTACGCACTTGCTTACTAATGTGGGCGGTAAACGTACTAT<br>GACTGCAGCTGGCCCGACGCGTAGCTTCGGACCCGCCCCGGAAGGGACTATCGTGACTGAGCC<br>TCTTCCGGAGGTGCATACCGAGCATCGCAATTACTTTGAGAATTATATCCGTGCGTACTATGGCGA<br>AGAGGAGTTTTATGTTAAAATTCCCGAGGTTAAGCGCGTACTTAAGTTAATGGACGCCGTTCGTG<br>AAAGCGGCCGCACGGGCAAATCAATTGACTTCGAG

Supplementary Figures

a

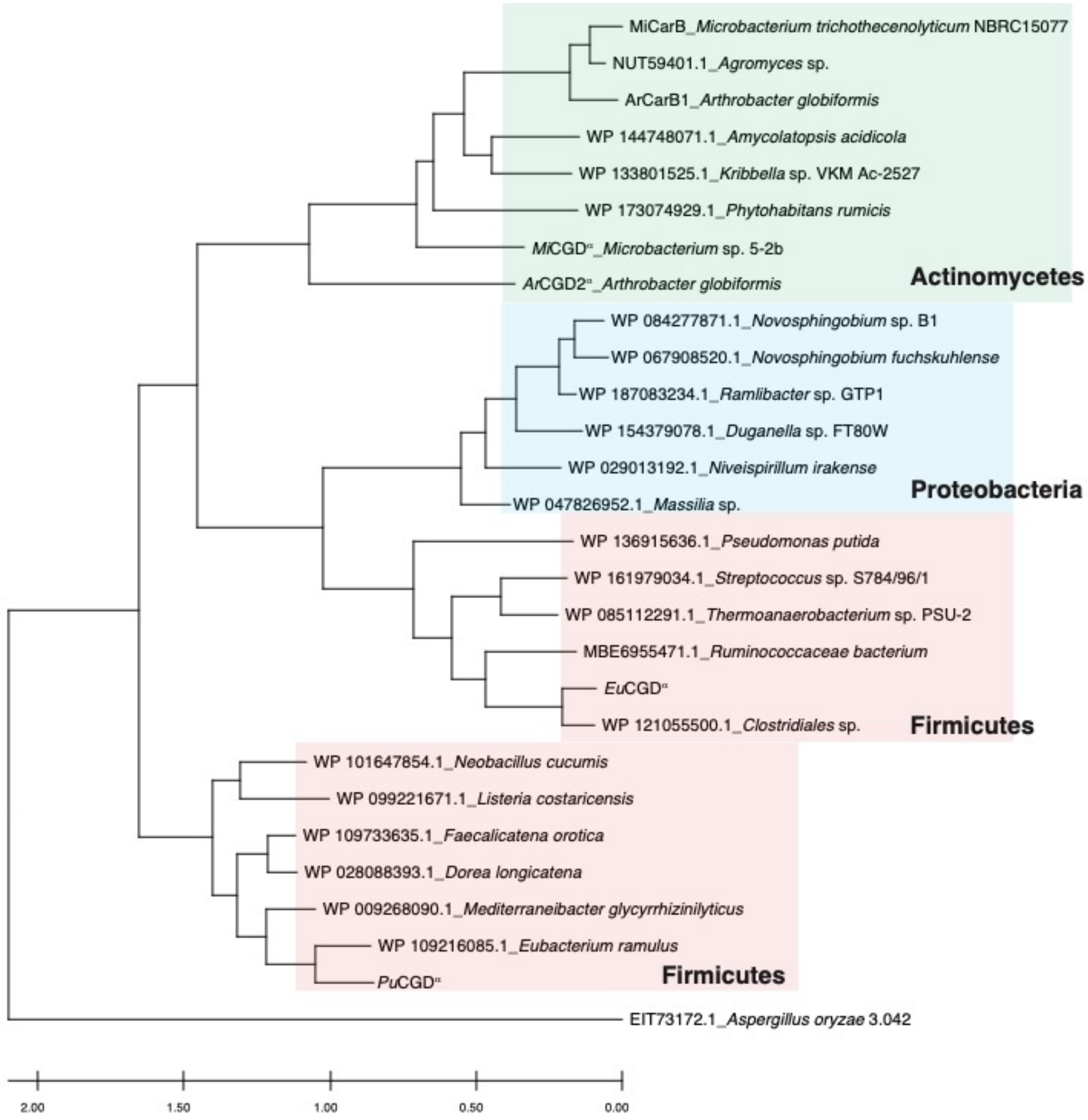

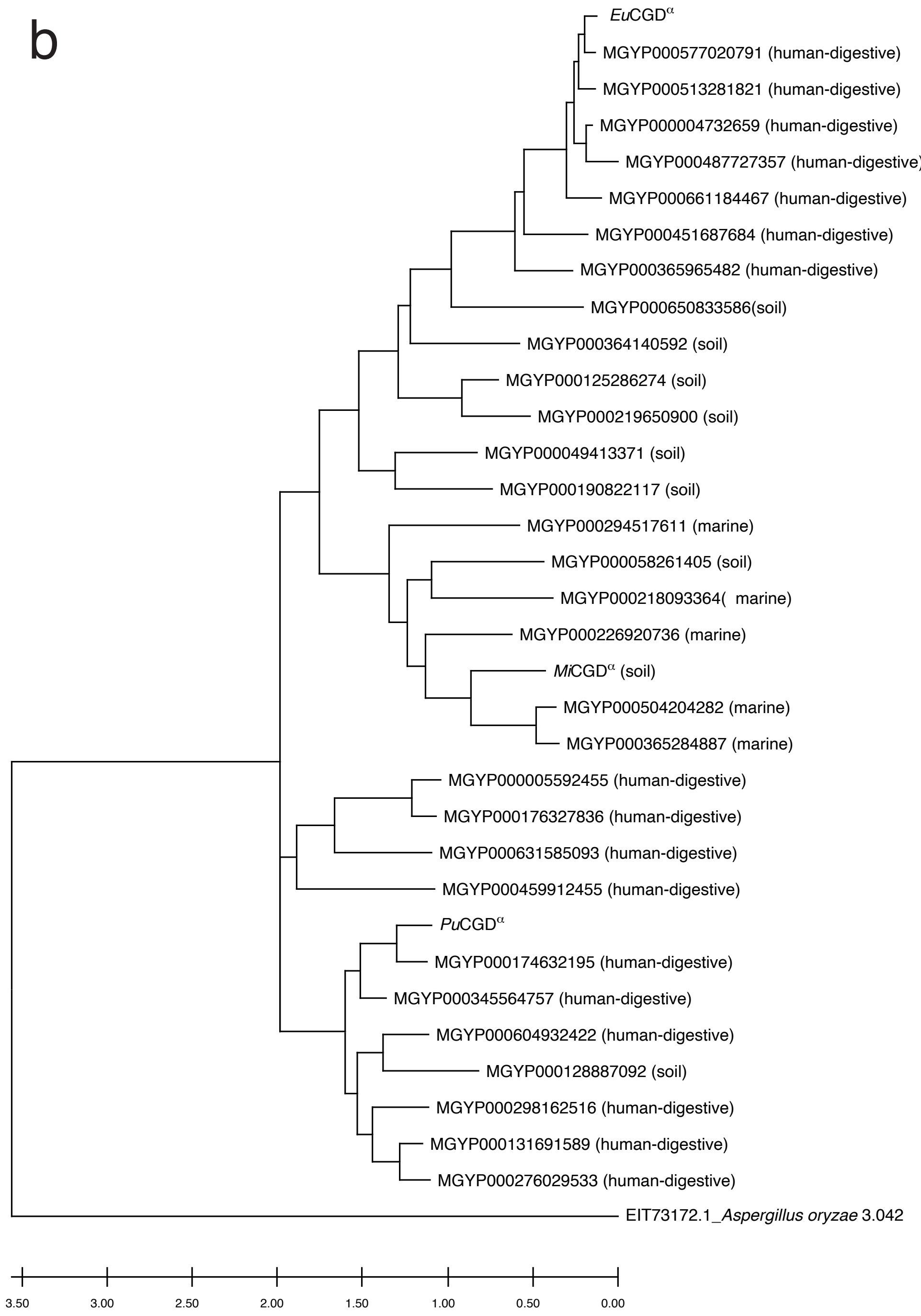

Supplementary Figure 1. Phylogenetic analyses of *Pu*CGD and its homologs.

(a) Phylogenetic analysis with the enzymes identified in a search of the National Center for Biotechnology Information (NCBI) database. The amino acid sequences were prepared by BLAST analyses, using *Pu*CGD$^{\alpha}$, *Eu*CGD$^{\alpha}$, and *Mi*CGD$^{\alpha}$ as queries. The proteins with more than 45% similarity with *Pu*CGD$^{\alpha}$ were used for the phylogenetic analysis. (b) Phylogenetic analysis with the enzymes from metagenomic data. The amino acid sequences were prepared by a similarity analysis from the MGnify protein sequence database (https://www.ebi.ac.uk/metagenomics/), using *Pu*CGD$^{\alpha}$, *Eu*CGD$^{\alpha}$, and *Mi*CGD$^{\alpha}$, as queries. Sugar isomerase from *Aspergillus oryzae* was used as an outer group. The *Pu*CGD homologs are widely distributed in both aerobic and anaerobic Actinobacteria, Proteobacteria, and Firmicutes species from soil, marine environments, and the human digestive system.

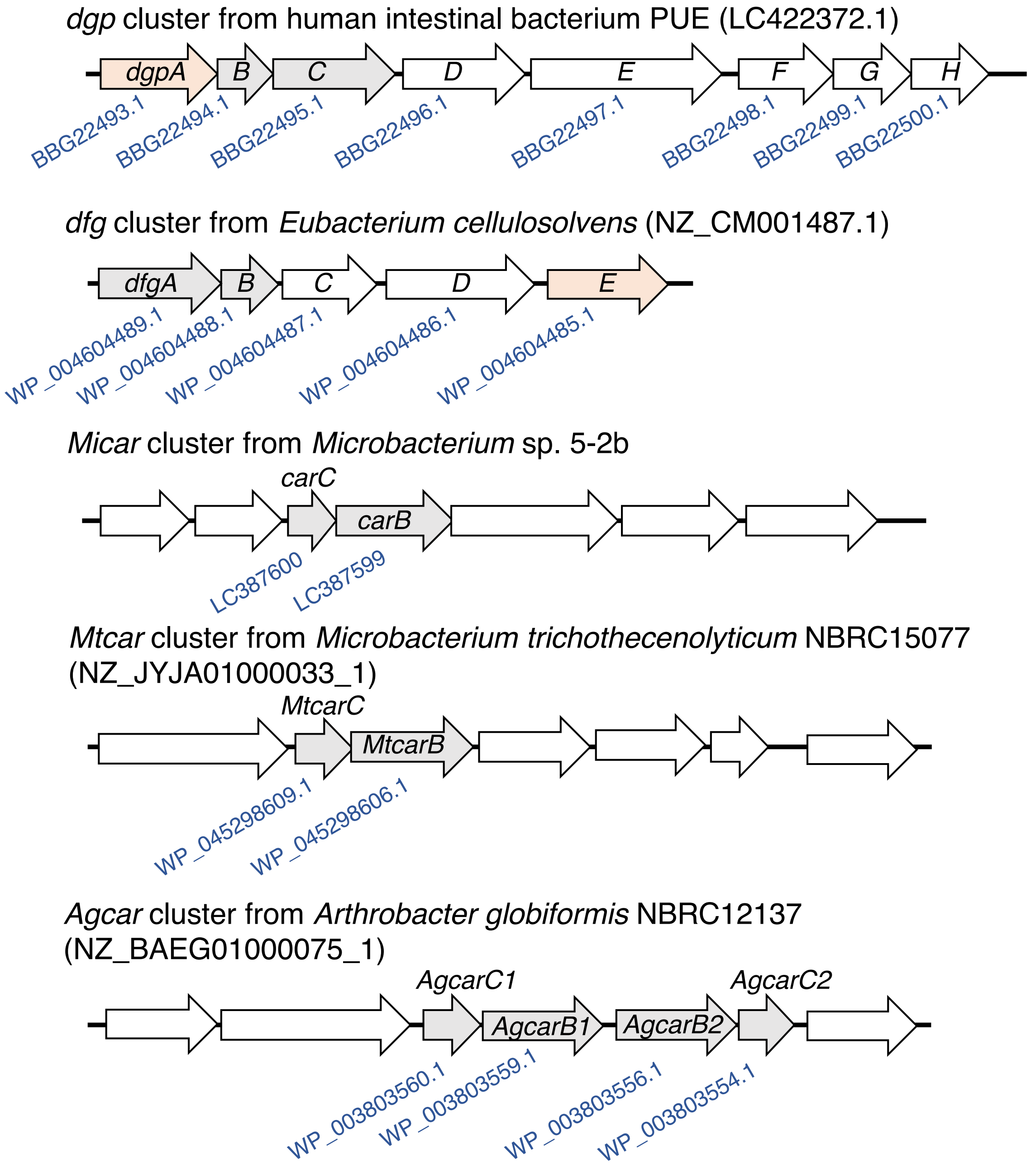

Supplementary Figure 2. Gene cluster analysis of *Pu*CGD homologs.

Analyses of gene clusters of various bacteria that contain a DgpB (*Pu*CGD$^{\alpha}$) homologous enzyme gene. The genes of *C*-deglycosylases are colored gray and the genes of oxidoreductase enzymes are colored orange. The homologous genes of DgpC are located next to DgpB homologous genes in the genomes.

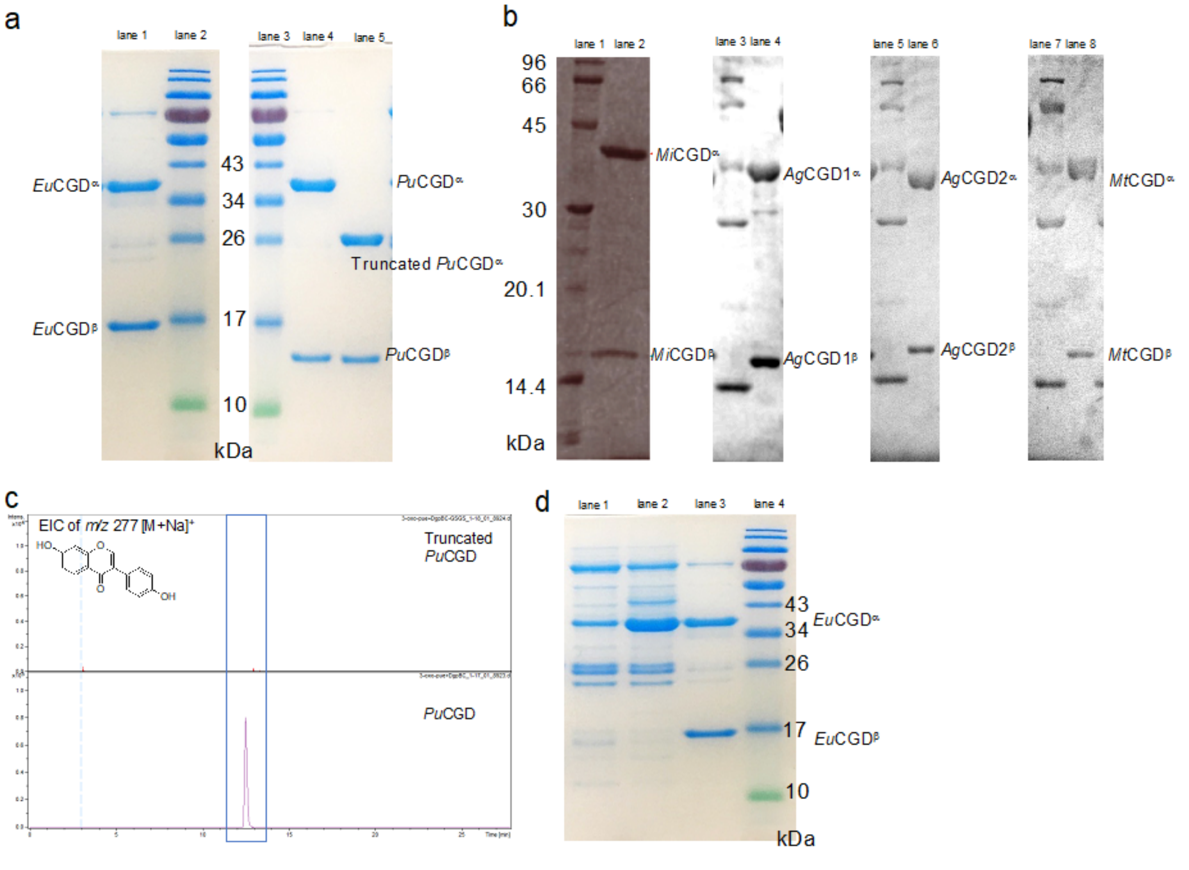

Supplementary Figure 3. Purification and gel-filtration analysis of C-deglycosylation enzymes. (a) SDS-PAGE analysis of purified *Eu*CGD (DgpB-C), *Pu*CGD (DfgA-B), and truncated *Pu*CGD (lane 1, purified *Eu*CGD; lanes 2 and 3, standard markers (PageRuler Prestained Protein Ladder (Thermo Fisher Scientific); lane 4, purified *Pu*CGD; and lane 5, N179-D244 lid domain truncated *Pu*CGD). (b) SDS-PAGE of purified *Mi*CGD (*Mi*CGD$^{\alpha}$: CarB, and *Mi*CGD$^{\beta}$: CarC), *Ag*CGD1, *Ag*CGD2, and *Mt*CGD (lanes 1, 3, 5 and 7; standard markers (LMW Marker Kit (GE Healthcare)); lane 2, purified *Mi*CGD; lane 4, purified *Ag*CGD1; lane 6, *Ag*CGD2; and lane 8, *Mt*CGD). (c, d) Activity of the N179-D244 lid domain-truncated *Pu*CGD. (c) Extracted ion chromatograms (m/z=277 [M+Na]$^{+}$) of the reaction mixture with truncated *Pu*CGD and the reaction mixture incubated with wild type *Pu*CGD. (d) SDS-PAGE of swapped complexes (lane 1, *Pu*CGD$^{\alpha}$-*Eu*CGD$^{\beta}$; lane 2, *Eu*CGD$^{\alpha}$-*Pu*CGD$^{\beta}$; lane 3, *Pu*CGD; and lane 4, standard markers). Similar results of SDS-PAGE were obtained at least three times.

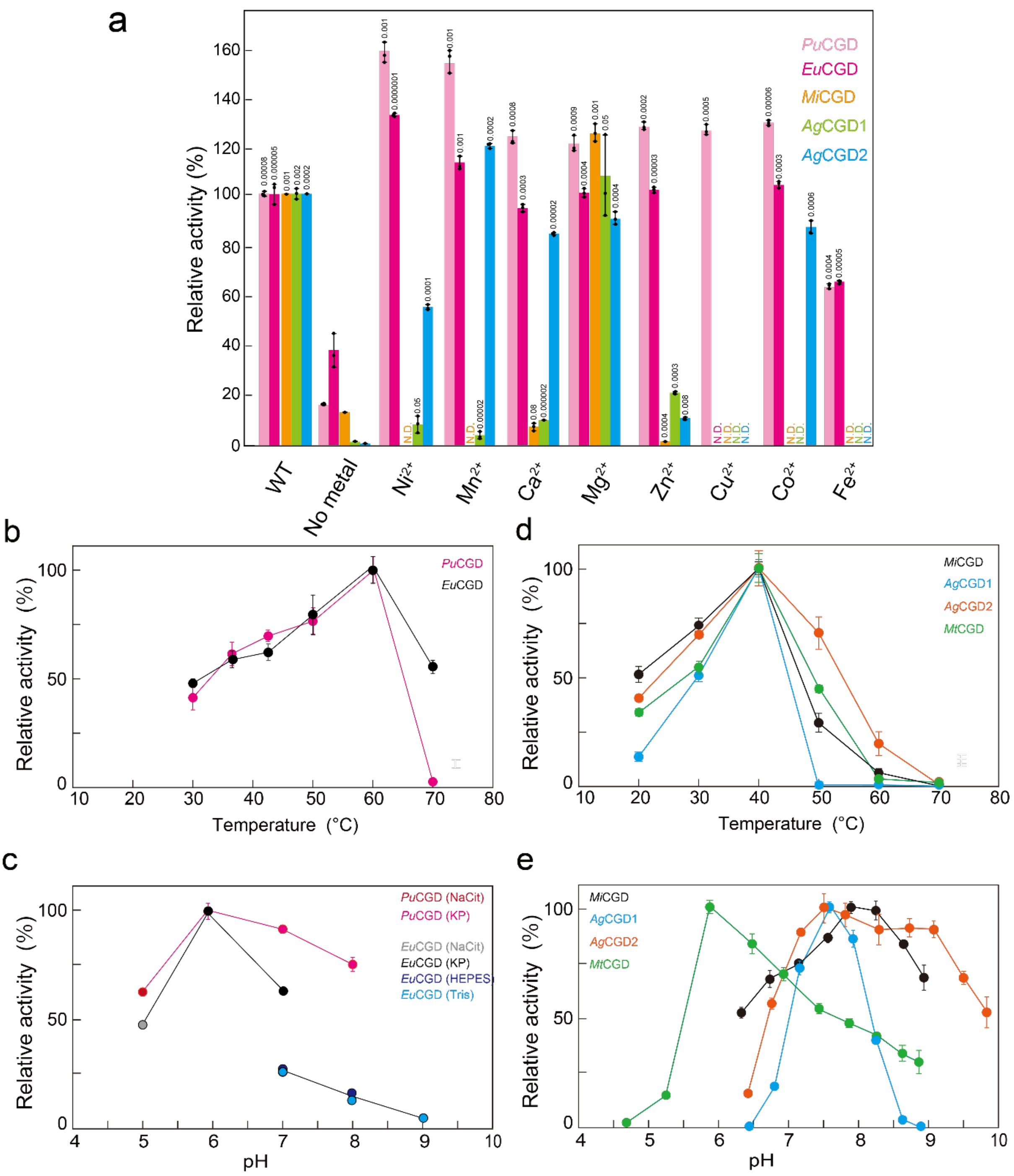

Supplementary Figure 4. Metal-dependency and optimization of C-deglycosylation reaction.

(a) Effects of metal ions on the enzyme reactions of C-deglycosylation enzymes. (b-e) Temperature and pH optimization of (b, c) the enzymes from intestinal bacteria and (d, e) the enzymes from soil bacteria. Statistical comparisons between means for the "No metal", which is the chelate reagent-treated enzyme and WT (wild type) and each mutant were performed by Student's t-test (2-tailed): *p*-values were shown over each bar. The bars are means of n = 3 independent experiments and error bars indicate standard deviations. Data are presented as mean values +/- SD. All experiments were repeated independently three times with similar results.

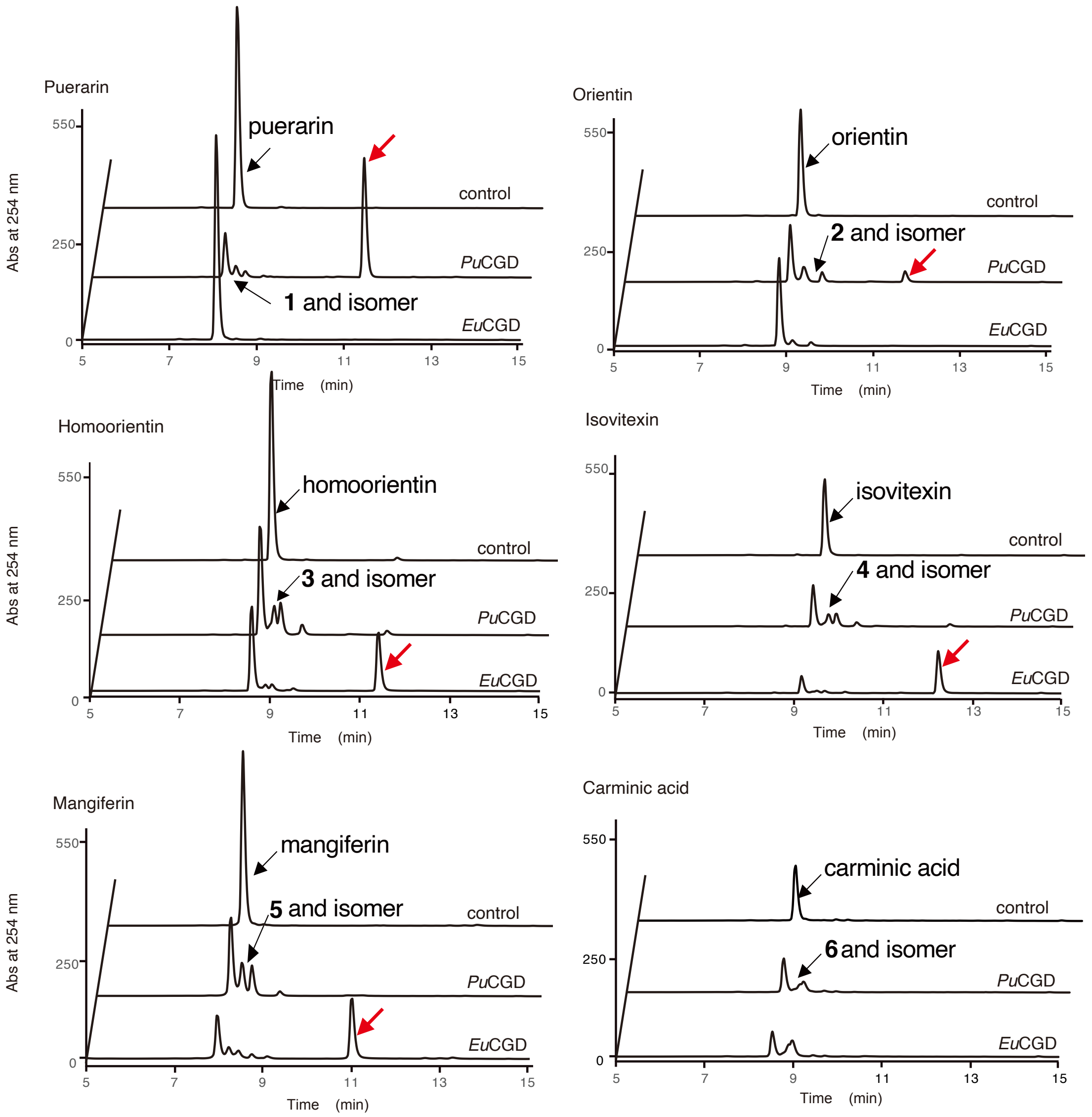

Supplementary Figure 5. HPLC analysis of the enzyme reaction products of CGDs from intestinal bacteria.

HPLC chromatograms of the C-deglycosylation reactions performed with enzymes from intestinal microorganisms. Red arrows indicate the production of aglycones.

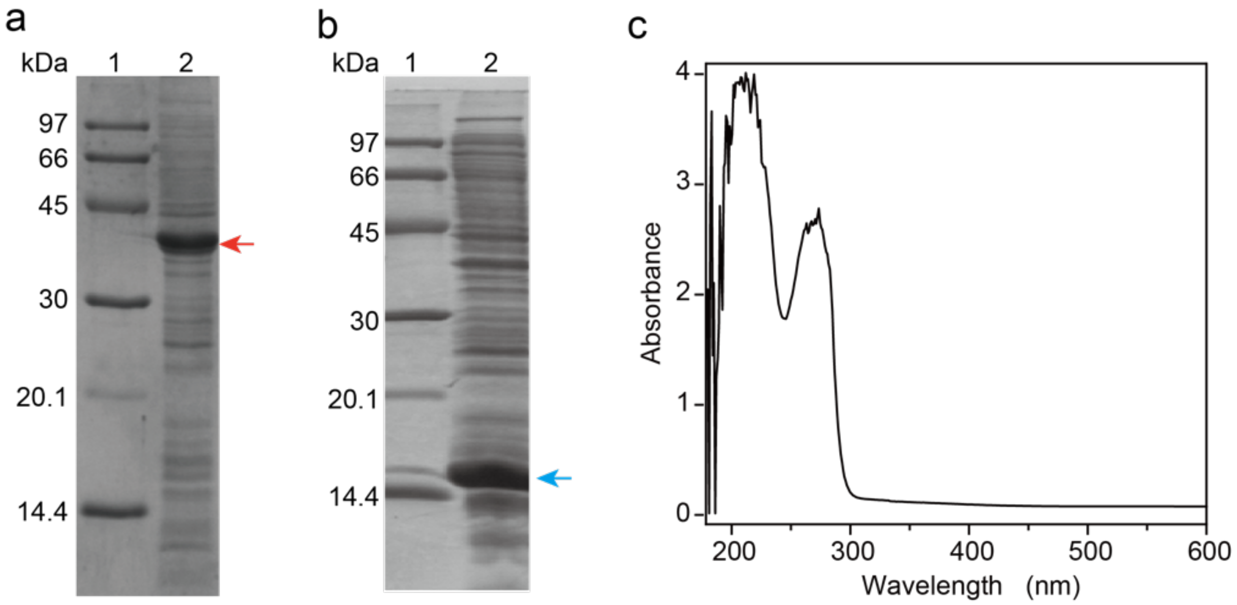

Supplementary Figure 6. SDS-PAGE of CarB (α- and β-subunit of *Mi*CGD) and UV-Vis spectrum of *Mi*CGD expressed in *E. coli* Rosetta2 (DE3).

(a) Lane 1, protein markers; lane 2, cell-free extract of *E. coli* Rosetta2 (DE3)-expressed CarB. The red arrow indicates CarB. (b) Lane 1, protein markers; lane 2, the cell-free extract of *E. coli* Rosetta2 (DE3)-expressed CarC. The light blue arrow indicates CarC. (c) UV-Vis spectrum of the purified CarB-C complex, which was co-expressed in *E. coli* Rosetta2 (DE3). Similar results of SDS-PAGE were obtained at least three times.

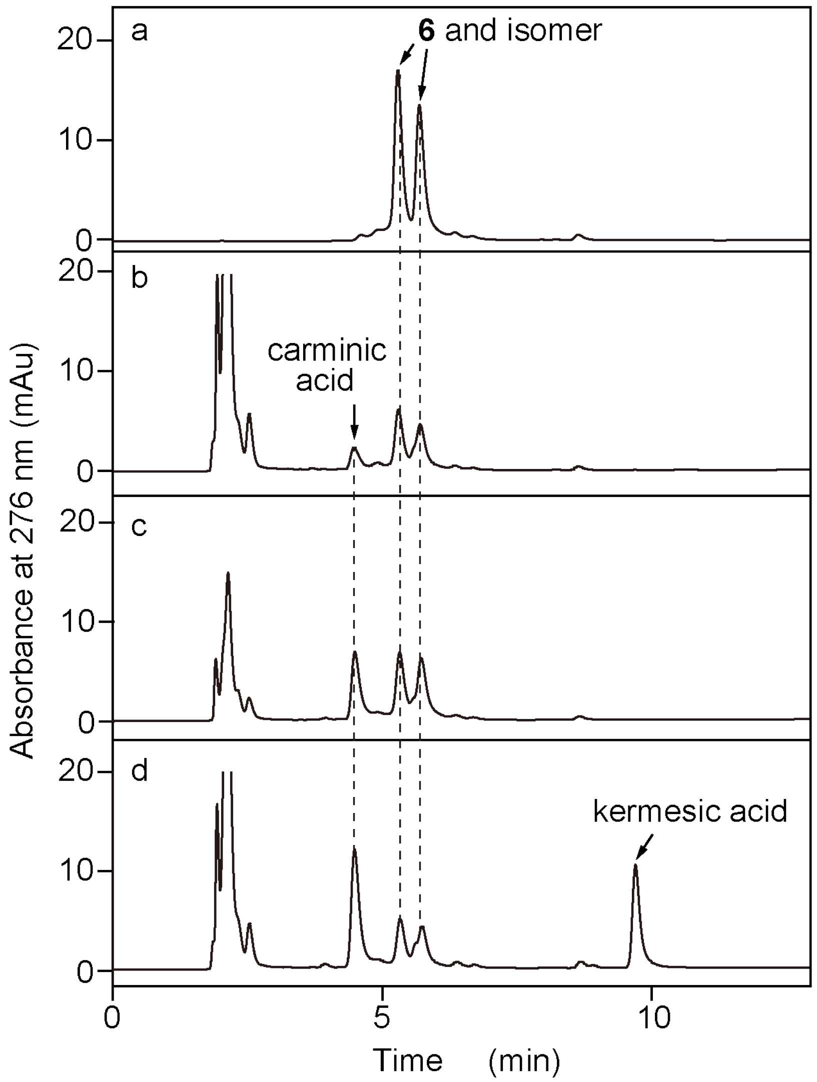

Supplementary Figure 7. HPLC chromatograms of reaction mixtures after incubation of CarB and/or CarC with 3'-oxo-carminic acid.

3'-oxo-carminic acid (**a**), and the 3'-oxo-carminic acid incubated with CarB (**b**), with CarC (**c**), and with CarB and CarC (**d**). The arrows indicate carminic acid, 3'-oxo carminic acid **6** and its isomer, kermesic acid.

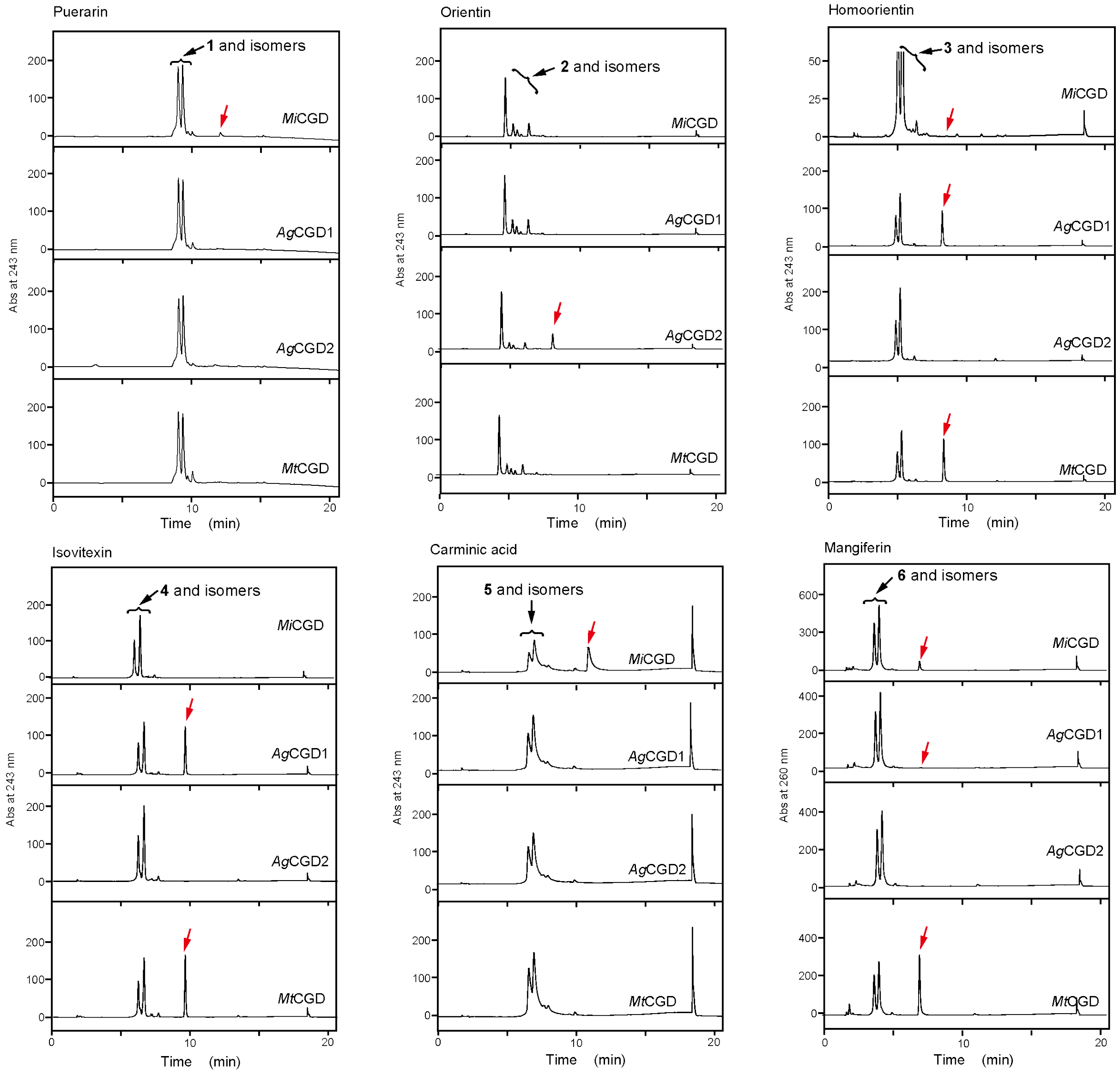

Supplementary Figure 8. HPLC analysis of the enzyme reaction products of CGDs from soil bacteria.

HPLC chromatograms for the *C*-deglycosylation reactions performed with enzymes from soil microorganisms. Red arrows indicate the production of aglycones.

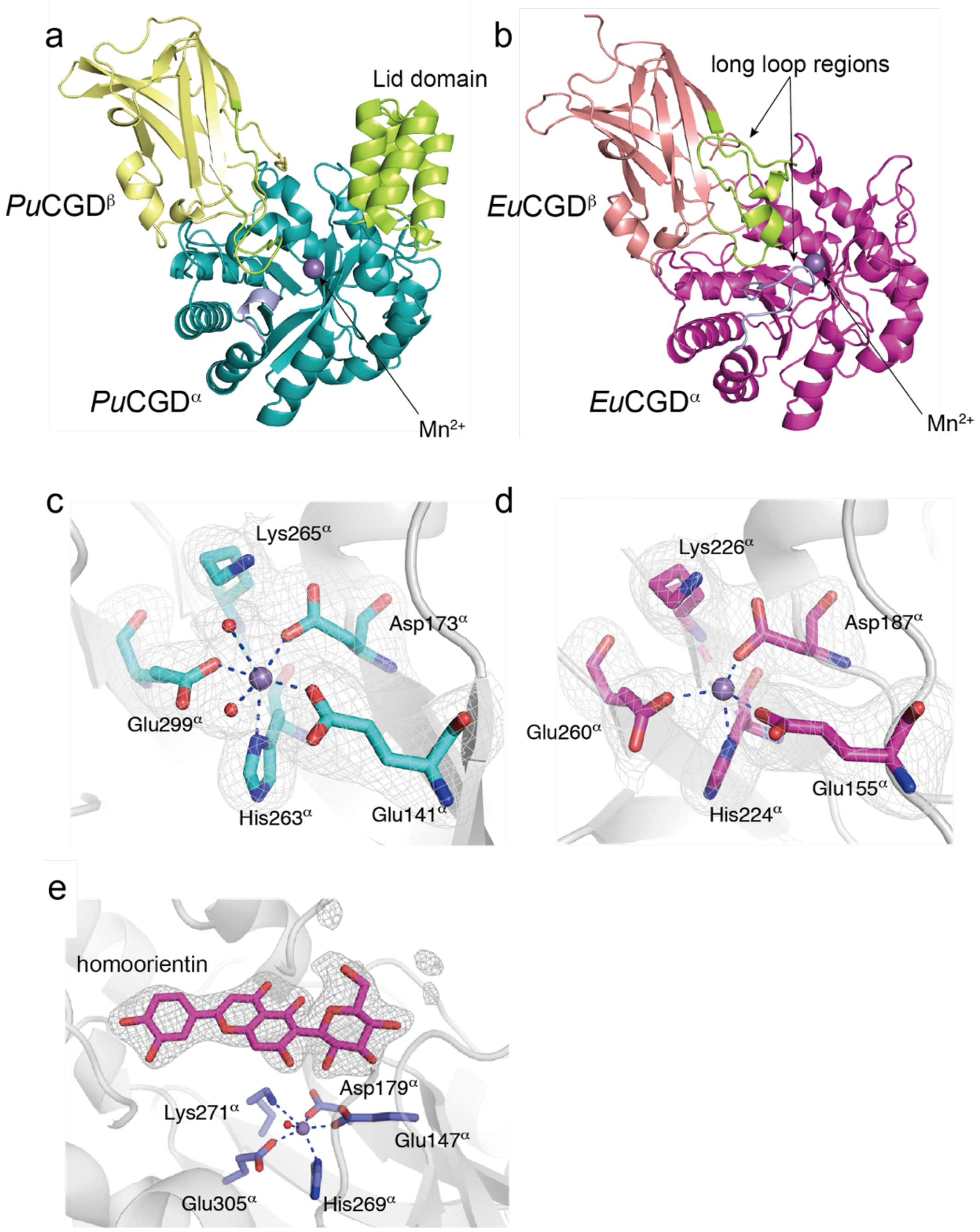

Supplementary Figure 9. Structures of *Pu*CGD, *Eu*CGD, and *Ag*CGD2 (related to Figure 2).

(a, b) The heterodimer structures of (a) *Pu*CGD and (b) *Eu*CGD. β-Sandwich structures of *Pu*CGD$^{\beta}$ and *Eu*CGD$^{\beta}$, shown in light yellow and pink, respectively, and TIM barrel structures of *Pu*CGD$^{\alpha}$ and *Eu*CGD$^{\alpha}$, shown in cyan and magenta, respectively. The lid domains (*Pu*CGD$^{\alpha}$) and long loop regions (M121-N133 in *Eu*CGD$^{\alpha}$ and T110-V139 in *Eu*CGD$^{\beta}$) of *Eu*CGD are colored light green and light blue, respectively. Corresponding regions in *Pu*CGD are shown in the same colors. (c, d) Fo-Fc polder omit maps of the metal-binding sites of (c) *Pu*CGD and (d) *Eu*CGD. The electron density maps of the ligands are represented by a gray mesh, contoured at +4.0 sigma. The coordination of the manganese ion is represented by dashed blue lines. (e) Fo-Fc polder omit map of homoorientin in the structure of *Ag*CGD2. The electron density map of the ligands is represented by a gray mesh, contoured at +5.0 sigma.

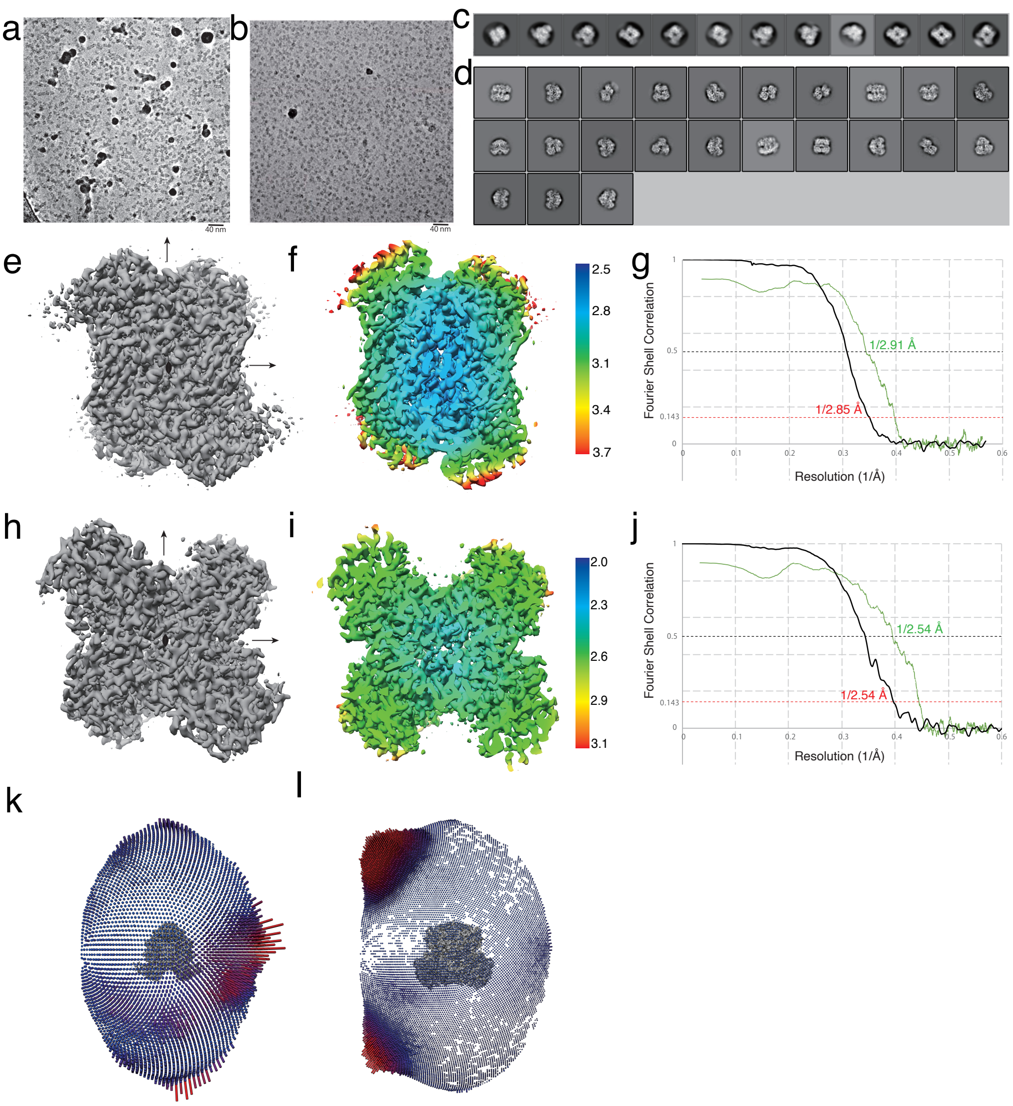

Supplementary Figure 10. Cryo-EM analysis of *Pu*CGD and *Eu*CGD.

(a and b) Representative cryo-EM micrographs of (a) *Pu*CGD and (b) *Eu*CGD particles. Similar micrographs were obtained two times. (c and d) 2D class averages of (c) *Pu*CGD and (d) *Eu*CGD particle images. (e) Cryo-EM map of *Pu*CGD. (f) Local resolution of the reconstructed density map of *Pu*CGD. (g) Half-map FSC (black) and model-map FSC (green) curves of the *Pu*CGD cryo-EM maps. The horizontal dashed lines represent the 0.5 (black) and 0.143 (red) cutoff values, respectively. The gold-standard FSC curves between the two half maps with the indicated resolutions at FSC = 0.143 are colored red. The FSC curves between the atomic model and the final map with the indicated resolution at FSC = 0.5 are colored green. (h) Cryo-EM map of *Eu*CGD. (i) Local resolution of the reconstructed density map of *Eu*CGD. (j) Half-map FSC (black) and model-map FSC (green) curves of *Eu*CGD cryo-EM maps. The horizontal dashed lines represent the 0.5 (black) and 0.143 (red) cutoff values, respectively. The gold-standard FSC curves between the two half maps with the indicated resolution at FSC = 0.143 are colored red. The FSC curves between the atomic model and the final map with the indicated resolution at FSC = 0.5 are colored green. The complete workflow for cryo-EM data-processing of *Pu*CGD and *Eu*CGD is shown in the *Supplementary Methods*. (k and l) The sharpened density map of the final reconstruction and the angular distributions of (k) *Pu*CGD and (l) *Eu*CGD.

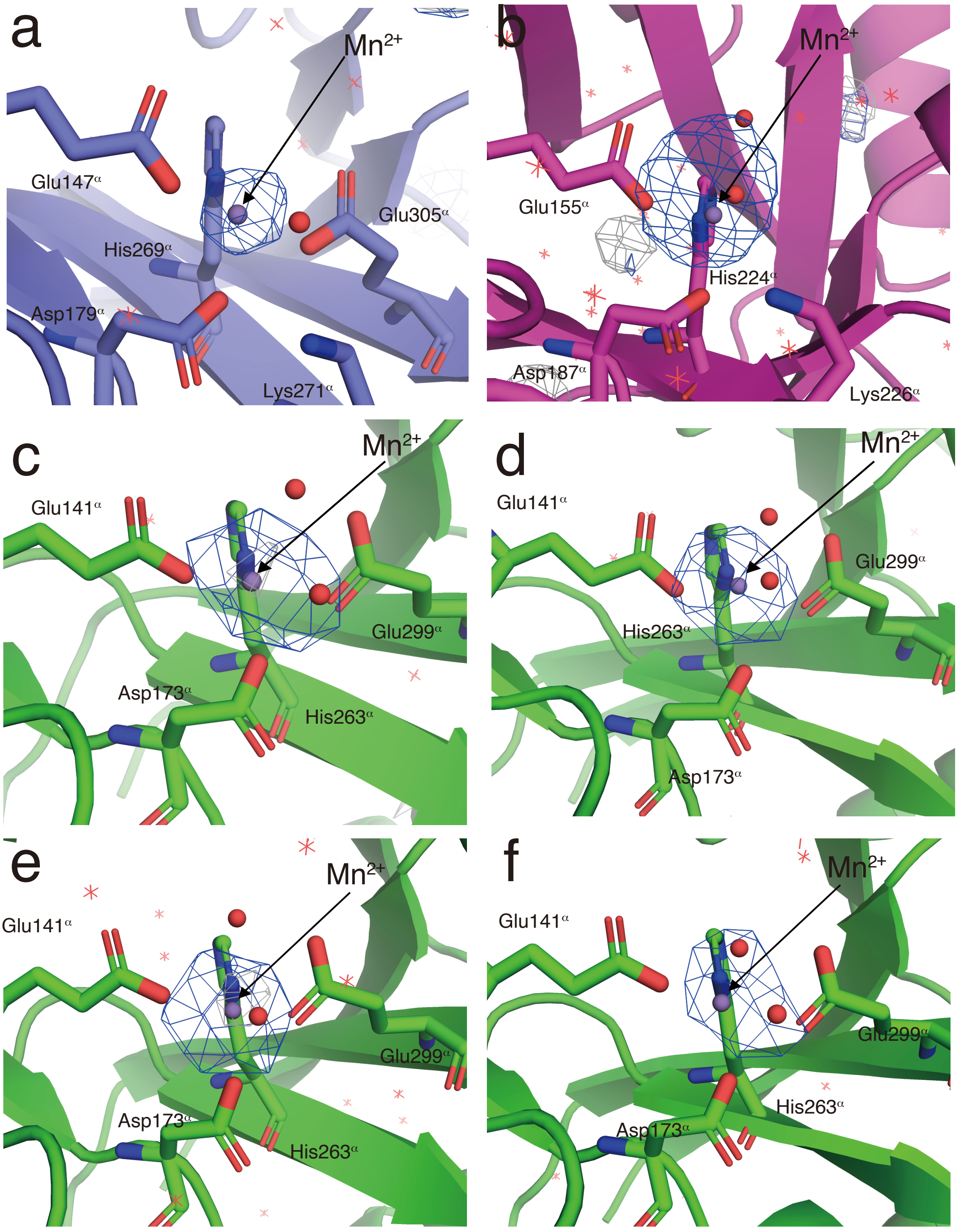

Supplementary Figure 11. Anomalous difference Fourier map.

Anomalous difference Fourier map for the Mn ion of (a) *Ag*CGD2, (b) *Eu*CGD, (c-f) *Pu*CGD (monomers A (c), C (d), E (e), and G (f) of *Pu*CGD). This map was calculated using the diffraction data collected at the peak wavelength (1.8900 Å) of manganese. The map is contoured at 5 σ level.

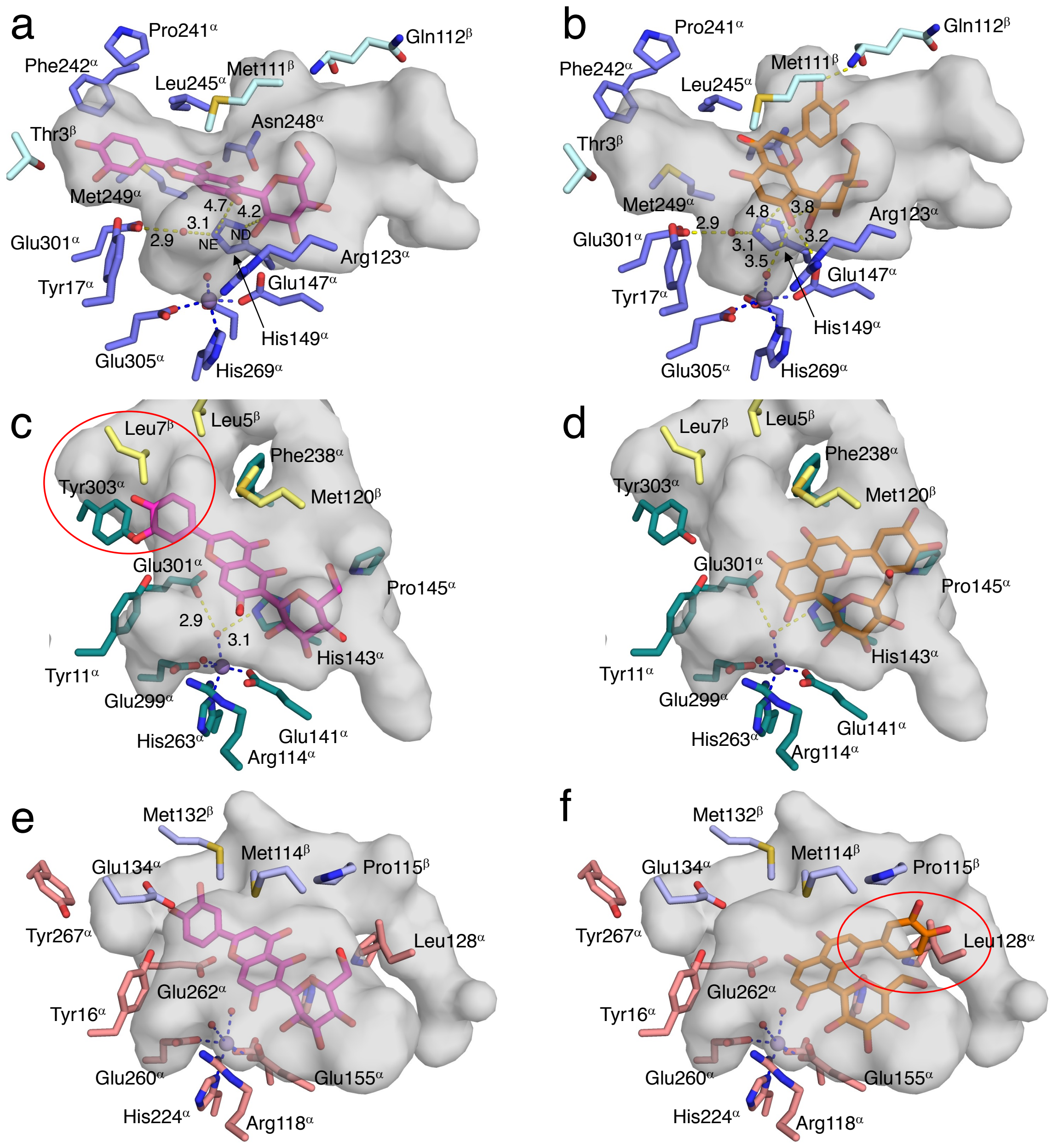

Supplementary Figure 12. The cocrystal structure and manually docking models of homoorientin and orientin in CGDs.

(a) The crystal structure of *Ag*CGD2 with homoorientin, (b) the docking model of *Ag*CGD2 with orientin, (c, d) the docking model of *Pu*CGD2 with (c) homoorientin and (d) orientin. (e and f) The docking model of *Eu*CGD2 with (e) homoorientin and (f) orientin. Side views of the active site cavities are depicted as gray surfaces. The hydrogen bonds are shown by dashed yellow lines. The red circles show the steric hindrance between active site residues and docked ligands.

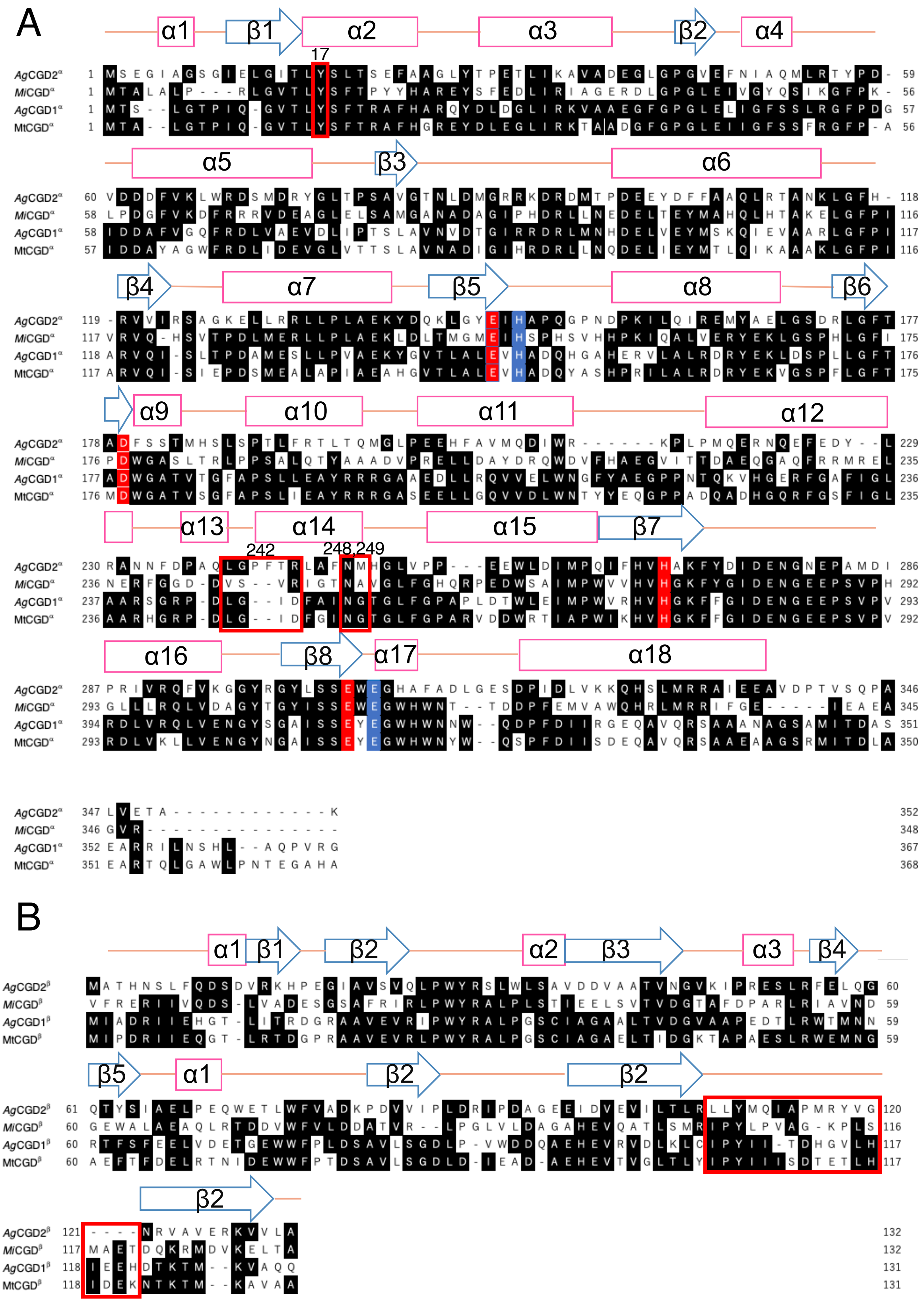

Supplementary Figure 13. Sequence alignments of CGDs α-subunit and β-subunit.

The metal-coordinating residues and mutated His149$^{\alpha}$ and Glu307$^{\alpha}$ are highlighted in red and blue in panel A, respectively. The important regions for substrate specificity, described in the main text, are highlighted in red boxes.

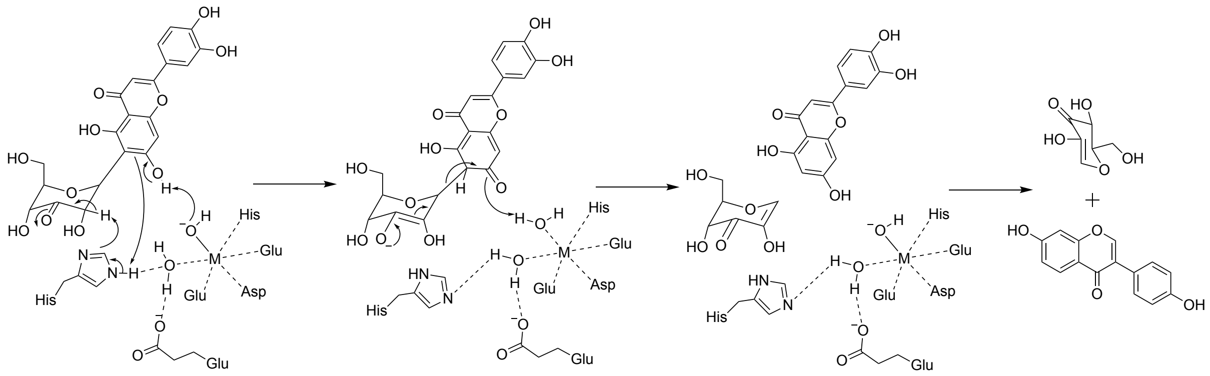

Supplementary Figure 14. **Proposed mechanism of *C*-deglycosylation reaction for C6-glucosides.** M represents a metal ion.

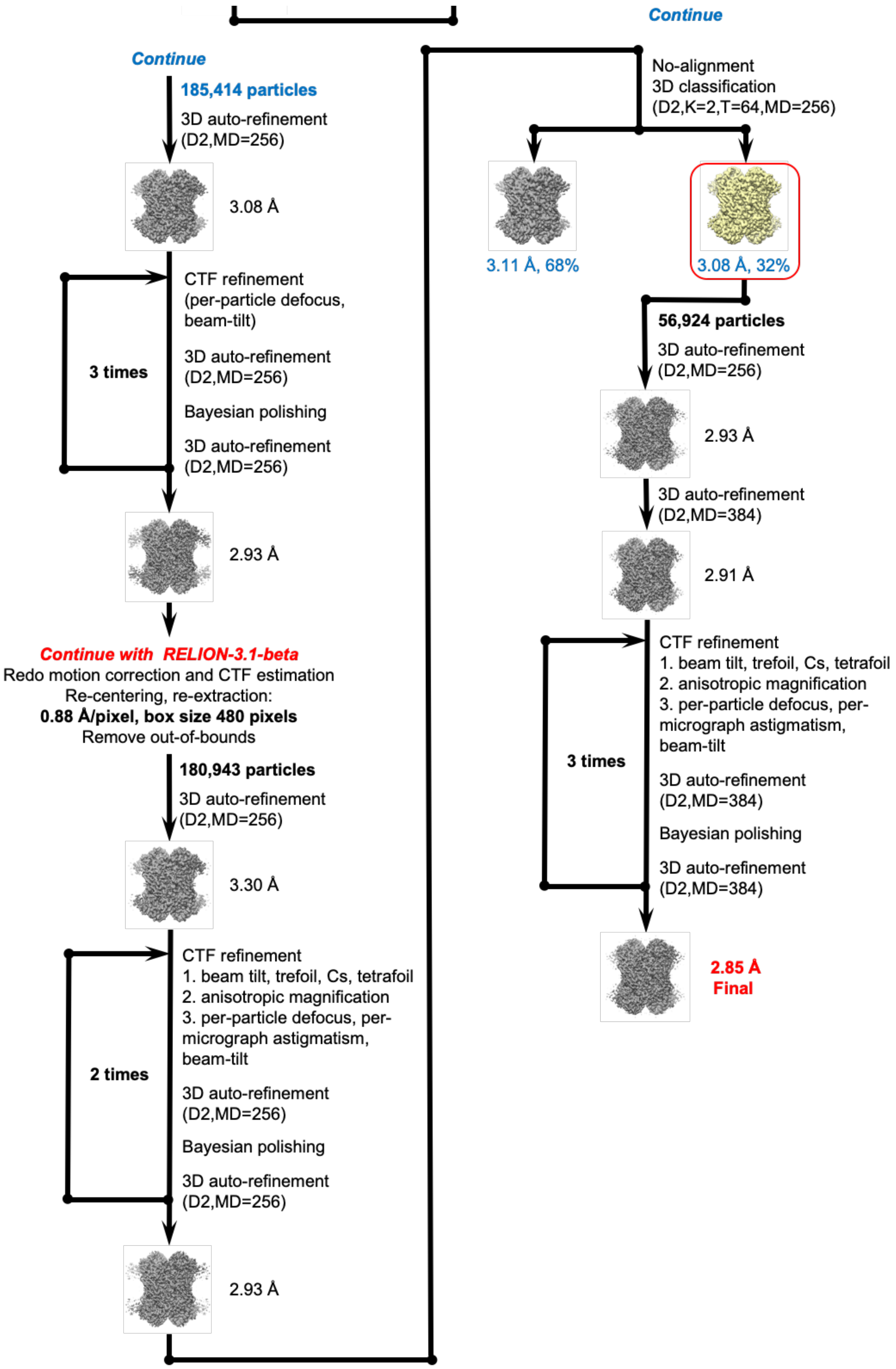

Continue
185,414 particles
3D auto-refinement
(D2,MD=256)
3.08 Å
CTF refinement
(per-particle defocus,
beam-tilt)
3 times
3D auto-refinement
(D2,MD=256)
Bayesian polishing
3D auto-refinement
(D2,MD=256)
2.93 Å
Continue with RELION-3.1-beta
Redo motion correction and CTF estimation
Re-centering, re-extraction:
0.88 Å/pixel, box size 480 pixels
Remove out-of-bounds
180,943 particles
3D auto-refinement
(D2,MD=256)
3.30 Å
CTF refinement
1. beam tilt, trefoil, Cs, tetrafoil
2. anisotropic magnification
3. per-particle defocus, per-micrograph astigmatism, beam-tilt
2 times
3D auto-refinement
(D2,MD=256)
Bayesian polishing
3D auto-refinement
(D2,MD=256)
2.93 Å
Continue
No-alignment
3D classification
(D2,K=2,T=64,MD=256)
3.11 Å, 68%
3.08 Å, 32%
56,924 particles
3D auto-refinement
(D2,MD=256)
2.93 Å
3D auto-refinement
(D2,MD=384)
2.91 Å
CTF refinement
1. beam tilt, trefoil, Cs, tetrafoil
2. anisotropic magnification
3. per-particle defocus, per-micrograph astigmatism,
beam-tilt
3 times
3D auto-refinement
(D2,MD=384)
Bayesian polishing
3D auto-refinement
(D2,MD=384)
2.85 Å
Final

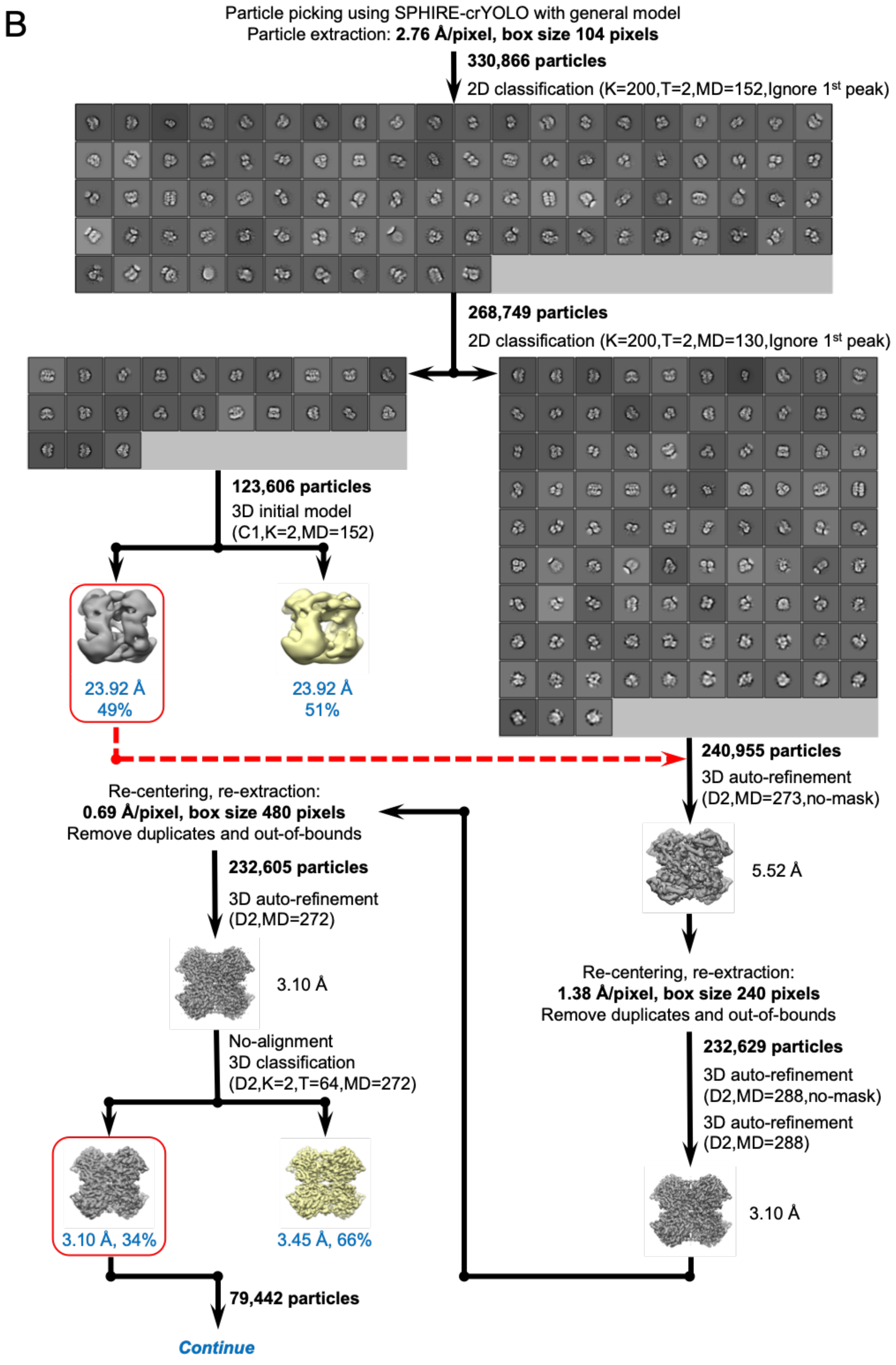
B
Particle picking using SPHIRE-crYOLO with general model
Particle extraction: 2.76 Å/pixel, box size 104 pixels
330,866 particles
2D classification (K=200,T=2,MD=152,Ignore 1st peak)
268,749 particles
2D classification (K=200,T=2,MD=130,Ignore 1st peak)
123,606 particles
3D initial model
(C1,K=2,MD=152)
23.92 Å
49%
23.92 Å
51%
240,955 particles
3D auto-refinement
(D2,MD=273,no-mask)
5.52 Å
Re-centering, re-extraction:
0.69 Å/pixel, box size 480 pixels
Remove duplicates and out-of-bounds
232,605 particles
3D auto-refinement
(D2,MD=272)
3.10 Å
No-alignment
3D classification
(D2,K=2,T=64,MD=272)
3.10 Å, 34%
3.45 Å, 66%
79,442 particles
Continue
Re-centering, re-extraction:
1.38 Å/pixel, box size 240 pixels
Remove duplicates and out-of-bounds
232,629 particles
3D auto-refinement
(D2,MD=288,no-mask)
3D auto-refinement
(D2,MD=288)
3.10 Å

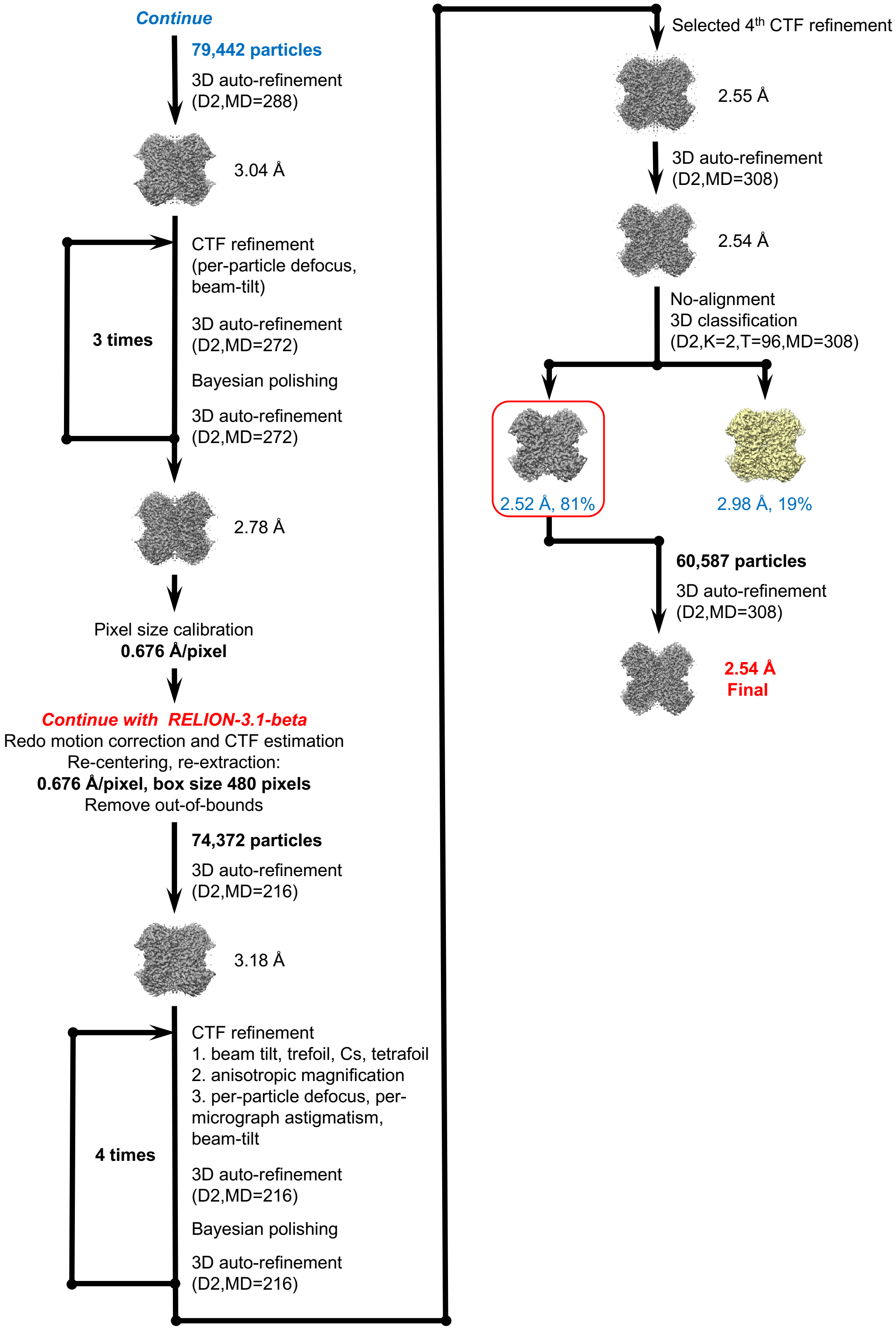

Supplementary Figure 15. Workflow for cryo-EM data-processing of DgpB-C and DfgA-B, and data statistics.

Workflow for (A) *Pu*CGD and (B) *Eu*CGD processings.

Supplementary Methods

Cryo-EM data processing of *Pu*CGD and *Eu*CGD (Supplementary Fig. 15)

The movie frames were aligned, dose-weighted, and averaged using Motioncor2, on 5 × 5 tiled frames with a B-factor of 200 applied, to correct for beam-induced specimen motion and to account for radiation damage by applying an exposure-dependent filter. The micrographs with total accumulated motion larger than 100 Å were discarded. For *Pu*CGD, the non-weighted movie sums were used for CTF estimation (512-pixel box size, 30 Å minimum resolution, 5 Å maximum resolution, 0.1 amplitude contrast) with Gctf, while the dose-weighted sums were used for all subsequent steps of image processing. Micrographs showing an obvious indication of ice crystallization (i.e. strong ice ring in the Fourier space) were manually discarded. The particles were picked fully-automatically using SPHIRE-crYOLO with the general model, by setting a 137-pixel box size and a selection threshold of 0.1. Micrographs with fewer than 130 picks were excluded. For EuCGD, the non-weighted movie sums were used for CTF estimation (512-pixel box size, 30 Å minimum resolution, 3 Å maximum resolution, 0.10 amplitude contrast) with Gctf and CTFFIND4, while the dose-weighted sums were used for all subsequent steps of image processing. The images with Gctf's CTF max resolution better than 4 Å were selected. Micrographs showing an obvious indication of ice crystallization (i.e., strong ice ring in the Fourier space) were manually discarded. The particles were picked fully-automatically using SPHIRE-crYOLO with the general model, using the selection threshold of 0.3. Micrographs that contained fewer than 94 picks were excluded.

RELION3 was used in the following processing. For *Pu*CGD, a stack of 857,817 particle images was extracted from 1,963 dose-weighted sum micrographs while rescaling to 3.52 Å/pixel with a 64-pixel box size, and then subjected to two consecutive runs of reference-free 2D classification (1st run: 200 expected classes, 155 Å mask diameter; 2nd run: 200 expected classes, 127 Å mask diameter). The 597,534 particles corresponding to the best 12 classes of the 2nd run that displayed secondary-structural elements and multiple views of *Pu*CGD were selected for *ab initio* reconstruction (asymmetry, 4 expected classes, 155 Å mask diameter). The generated *ab initio* map was imposed with D2 symmetry, low-pass filtered to 25 Å, and used as an initial model for the subsequent 3D classification (4 expected classes). The volume of the best 3D class with the highest resolution was then rescaled to 0.88 Å/pixel with a 320-pixel box size, low-pass filtered to 25 Å, and used as an initial reference map for the subsequent 3D refinement. The two 3D classes with obviously bad images containing non-targeted objects were removed, and the selected particle images were re-centered and re-extracted using the same rescale settings. The particle images that became duplicated as a result of alignments and were not fully inside of the micrograph boundary anymore due to the changed box size were excluded. A total of 508,886 selected particles were 3D auto-refined (D2 symmetry, 178 Å mask diameter, no padding) twice, with the 1st run without a 3D mask and the 2nd with a soft-edged 3D mask created from the initial 3D reference of the 1st run (5-pixel extension, 10-pixel soft cosine edge). The 2nd run

resulted in the resolution of 3.31 Å. To refine the per-particle defocus, beam tilt, and beam-induced motion corrections, the cycle of CTF refinement and Bayesian polishing was repeated three times. To measure the degree of the improvement, 3D refinement (D2 symmetry, 178 Å mask diameter, no padding), with the volume of the previous run as the initial 3D reference, a soft-edged 3D mask created from the initial reference (5-pixel extension, 10-pixel soft cosine edge) and the solvent-flattened FSCs options, was used after each CTF refinement and Bayesian polishing step. The 3D refinement after the 3rd Bayesian polishing run yielded the resolution of 3.16 Å.

At this point, 457,708 selected particles were again re-centered and re-extracted with a 486-pixel box size (0.88 Å/pixel), followed by the removal of duplicated and out-of-bounds particles. Accordingly, the density map obtained by the 3D refinement after the 3rd Bayesian polishing run was also padded to the same box size, low-pass filtered to 15 Å, and used as an initial reference map for the subsequent 3D refinement (D2 symmetry, 256 Å mask diameter, no padding) with a soft-edged 3D mask (5-pixel extension, 10-pixel soft cosine edge). To improve the homogeneity of the particle stack, no-alignment 3D classification was conducted by setting the expected classes to 2 and the regularization parameter T to 8 (D2 symmetry, 256 Å mask diameter, no padding) with a soft-edged 3D mask (5-pixel extension, 10-pixel soft cosine edge), and 185,414 particles were selected by choosing the 3D class with higher resolution. The subsequent 3D refinement (D2 symmetry, 256 Å mask diameter, no padding) with a soft-edged 3D mask (5-pixel extension, 10-pixel soft cosine edge) generated 3.08 Å resolution. Three cycles of CTF refinement and Bayesian polishing were then executed as above, and the resolution improved to 2.93 Å.

For *Eu*CGD, a stack of 330,866 particle images was extracted from 1,614 dose-weighted sum micrographs while rescaling to 2.76 Å/pixel with a 104-pixel box size, and subjected to two consecutive runs of reference-free 2D classification ignoring first peak of CTFs (1st run: 200 expected classes, 152 Å mask diameter; 2nd run: 200 expected classes, 130 Å mask diameter). For the *ab initio* reconstruction, 123,606 particles corresponding to the best 23 classes of the 2nd run that displayed secondary-structural elements and multiple views of *Eu*CGD were selected (asymmetry, 2 expected classes, 152 Å mask diameter), and for the subsequent 3D refinement, 240,955 particles were selected with more relaxed criteria. A generated *ab initio* map with no obvious signs of structural damage was selected, imposed with D2 symmetry, low-pass filtered to 15 Å, and used as an initial reference map for the 3D refinement (D2 symmetry, 273 Å mask diameter, with padding).

The refined volume was then rescaled to 1.38 Å/pixel with a 240-pixel box size, and low-pass filtered to 15 Å for the subsequent 3D refinement. Accordingly, the particle images were also re-centered and re-extracted using the same rescale settings, followed by the removal of duplicated and out-of-bounds particles. Afterwards, 232,629 selected particles were 3D auto-refined (D2 symmetry, 288 Å mask diameter, no padding) twice, with the 1st run without the 3D mask and the 2nd with a soft-edged 3D mask created from the refined 3D map of the 1st run (5-pixel extension, 10-pixel soft cosine edge). The resolution of the 2nd run was 3.10 Å.

The refined volume was rescaled to 0.69 Å/pixel with a 480-pixel box size, and low-pass filtered to 15 Å for the subsequent 3D refinement. Accordingly, 232,605 selected particles were again re-centered and re-extracted using the same rescale settings, followed by the removal of duplicated and out-of-bounds particles, and were 3D auto-refined (D2 symmetry, 272 Å mask diameter, no padding) with a soft-edged 3D mask (5-pixel extension, 10-pixel soft cosine edge). To improve the homogeneity of the particle stack, no-alignment 3D classification was conducted by setting the expected classes to 2 and the regularization parameter T to 64 (D2 symmetry, 272 Å mask diameter, no padding) with a soft-edged 3D mask (5-pixel extension, 10-pixel soft cosine edge), and 79,442 particles were selected by choosing the 3D class with higher resolution. The subsequent 3D refinement (D2 symmetry, 288 Å mask diameter, no padding) with a soft-edged 3D mask (5-pixel extension, 10-pixel soft cosine edge) generated 3.04 Å resolution. Three cycles of CTF refinement and Bayesian polishing were then executed, and the resolution improved to 2.78 Å.

To correct higher-order aberrations and anisotropic magnification, in addition to per-particle defocus, beam tilt, and beam-induced motion, RELION 3.1.0-beta was used from this point forward. The movie frames were aligned, dose-weighted, and averaged using RELION's own implementation of a MotionCor2-like algorithm on 5 × 5 tiled frames, with a B-factor of 200 applied. The CTF parameters of each non-weighted movie sum were estimated with CTFFIND4 with a 512-pixel box size, 30 Å minimum resolution, 3 Å maximum resolution and 0.1 amplitude contrast. The images with CTF max resolutions better than 4 Å were selected.

For *Pu*CGD, from 1,879 dose-weighted sum micrographs, 180,943 particles associated with the 2.93 Å refined map were re-centered and re-extracted with a 480-pixel box size. The refined map was low-pass filtered to 15 Å, and used as an initial reference map for the subsequent 3D refinement (D2 symmetry, 256 Å mask diameter, no padding) with a soft-edged 3D mask (5-pixel extension, 10-pixel soft cosine edge). The cycle of CTF refinement and Bayesian polishing was then repeated twice. Following the RELION 3.1.0 tutorial, CTF refinement at each cycle was executed three times: first to correct higher-order aberrations (beam tilt, trefoil astigmatism, Cs, and tetrafoil astigmatism), second to correct anisotropic magnification, and third for per-particle defocus and per-micrograph astigmatism. The 3D refinement after the 2nd Bayesian polishing run yielded the resolution of 2.93 Å.

To further improve the homogeneity of the particle stack, no-alignment 3D classification was again conducted by setting the expected classes to 2 and the regularization parameter T to 64 (D2 symmetry, 256 Å mask diameter, no padding) with a soft-edged 3D mask (5-pixel extension, 10-pixel soft cosine edge). By choosing the 3D class with higher resolution, 56,924 particles were selected. The selected volume was 3D auto-refined (D2 symmetry, no padding, with a soft-edged 3D mask) twice: the 1st run with a 256 Å mask diameter, and the 2nd with a 384 Å mask diameter. The 2nd run resulted in the resolution of 2.91 Å. Again, three cycles of CTF refinement and Bayesian polishing were executed as above and generated the final result of 2.85 Å.

For *Eu*CGD, a stack of 74,372 particle images associated with the 2.78 Å refined map was re-centered and re-extracted from 1,598 dose-weighted sum micrographs, while rescaling to the calibrated pixel size of 0.676 Å/pixel with a 480-pixel box size. Accordingly, the refined map was also rescaled with the same settings, low-pass filtered to 15 Å, and used as an initial map for the subsequent 3D refinement (D2 symmetry, 216 Å mask diameter, no padding) with a soft-edged 3D mask (5-pixel extension, 10-pixel soft cosine edge). The cycle of CTF refinement and Bayesian polishing was then repeated four times, as above. The 3D refinement after the 4th CTF refinement step yielded the best resolution of 2.55 Å during the cycles. The particle stack was further 3D auto-refined using a larger mask diameter (D2 symmetry, 308 Å mask diameter, no padding) with a soft-edged 3D mask (5-pixel extension, 10-pixel soft cosine edge), and the resolution of 2.54 Å was obtained. To improve the homogeneity of the particle stack, no-alignment 3D classification was conducted by setting the expected classes to 2 and the regularization parameter T to 96 (D2 symmetry, 308 Å mask diameter, no padding) with a soft-edged 3D mask (5-pixel extension, 10-pixel soft cosine edge), and 60,587 particles were selected by choosing the 3D class with higher resolution. The last 3D refinement (D2 symmetry, 308 Å mask diameter, no padding) with a soft-edged 3D mask (5-pixel extension, 10-pixel soft cosine edge) generated the final result of 2.54 Å.

The local resolution of the final reconstruction was estimated using RELION-3's own implementation.

The initial *Pu*CGD and *Eu*CGD models were built with the Map to Model program in PHENIX[1], using the cryo-EM maps and amino acid sequences. The model was then manually modified with Coot[2] and refined by using Real-space refinement in PHENIX[3]. The cryo-EM maps of *Pu*CGD and *Eu*CGD have been deposited in the Electron Microscopy Data Bank (EMDB-30808 and EMDB-30809, respectively). The coordinates of *Pu*CGD and *Eu*CGD have been deposited in the Protein Data Bank (7DRD and 7DRE, respectively). The statistics of EM data processing and refinement are summarized in Supplementaly Table 3. The local resolution maps were produced with the ResMap program[4].

Supplementary References:

1. Terwilliger, T. C., Adams, P. D., Afonine, P. V. & Sobolev, O. V. A fully automatic method yielding initial models from high-resolution cryo-electron microscopy maps. *Nat. Methods* **15**, 905-908 (2018).
2. Emsley, P. & Cowtan, K. Coot: Model-building tools for molecular graphics. *Acta Crystallogr. Sect. D Biol. Crystallogr.* **60**, 2126-2132 (2004).
3. Afonine, P. V. *et al.* Real-space refinement in PHENIX for cryo-EM and crystallography. *Acta Crystallogr. Sect. D Biol. Crystallogr.* **74**, 531-544 (2018).
4. Kucukelbir, A., Sigworth, F. J. & Tagare, H. D. Quantifying the local resolution of cryo-EM density maps, *Nat. Methods.* **11**, 63-65 (2014).
