# Supplementary material for: C-Glycoside metabolism in the gut and in nature: Identification, characterization, structural analyses and distribution of C-C bond-cleaving enzymes
Source: Nat Commun. 2021 Nov 2;12:6294. doi: 10.1038/s41467-021-26585-1 (PMC8563793; doi:10.1038/s41467-021-26585-1)
Supplement: Supplementary file 2 — Reporting summary [file 41467_2021_26585_MOESM2_ESM.pdf]

nature research

Corresponding author(s): Michihiko Kobayashi, Ikuro Abe, Toshiya Senda

Last updated by author(s): Oct 8, 2021

Reporting Summary

Nature Research wishes to improve the reproducibility of the work that we publish. This form provides structure for consistency and transparency in reporting. For further information on Nature Research policies, see Authors & Referees and the Editorial Policy Checklist .

Statistics

For all statistical analyses, confirm that the following items are present in the figure legend, table legend, main text, or Methods section.

n/a	Confirmed	
☐	☒	The exact sample size (*n*) for each experimental group/condition, given as a discrete number and unit of measurement
☐	☒	A statement on whether measurements were taken from distinct samples or whether the same sample was measured repeatedly
☐	☒	The statistical test(s) used AND whether they are one- or two-sided<br>*Only common tests should be described solely by name; describe more complex techniques in the Methods section.*
☒	☐	A description of all covariates tested
☐	☒	A description of any assumptions or corrections, such as tests of normality and adjustment for multiple comparisons
☐	☒	A full description of the statistical parameters including central tendency (e.g. means) or other basic estimates (e.g. regression coefficient) AND variation (e.g. standard deviation) or associated estimates of uncertainty (e.g. confidence intervals)
☐	☒	For null hypothesis testing, the test statistic (e.g. *F*, *t*, *r*) with confidence intervals, effect sizes, degrees of freedom and *P* value noted<br>*Give P values as exact values whenever suitable.*
☒	☐	For Bayesian analysis, information on the choice of priors and Markov chain Monte Carlo settings
☒	☐	For hierarchical and complex designs, identification of the appropriate level for tests and full reporting of outcomes
☒	☐	Estimates of effect sizes (e.g. Cohen's *d*, Pearson's *r*), indicating how they were calculated

Our web collection on statistics for biologists contains articles on many of the points above.

Software and code

Policy information about availability of computer code

Data collection	No software used
Data analysis	Prism 9, PHENIX-ver 1.19-4092-000, ccp4-7.1, XDS ver Jan 31 2020, Coot 0.9, PyMOL ver 2.0.6 , CLUSTAL W 2.0.12, MEGAX 11, EMAN2, UCSF chimera 1. 13.1

For manuscripts utilizing custom algorithms or software that are central to the research but not yet described in published literature, software must be made available to editors/reviewers. We strongly encourage code deposition in a community repository (e.g. GitHub). See the Nature Research guidelines for submitting code & software for further information.

Data

Policy information about availability of data

All manuscripts must include a data availability statement. This statement should provide the following information, where applicable:

- Accession codes, unique identifiers, or web links for publicly available datasets
- A list of figures that have associated raw data
- A description of any restrictions on data availability

Source data are provided with this paper. The data generated in this study are provided in the Supplementary Information/Source Data files. The crystallographic data that support the findings of this study are available from the Protein Data Bank (http://www.rcsb.org). The coordinates and the structure factor amplitudes for the apo structures of PuCGD, EuCGD, AgCGD2 and AgCGD2 complexed with homoorientin were deposited under accession code 7EXZ, 7EXB, 7DNM, and 7DNN, respectively. The cryo-EM maps and the atomic coordinates for PuCGD and EuCGD determined by cryo-EM have been deposited in Electron Microscopy Data Bank (EMDB, https://www.ebi.ac.uk/pdbe/emdb/) and PDB with accession codes EMD-30808 and EMD-30809, and 7DRD and 7DRE, respectively.

Field-specific reporting

Please select the one below that is the best fit for your research. If you are not sure, read the appropriate sections before making your selection.

- [x] Life sciences
- [ ] Behavioural & social sciences
- [ ] Ecological, evolutionary & environmental sciences

For a reference copy of the document with all sections, see nature.com/documents/nr-reporting-summary-flat.pdf

Life sciences study design

All studies must disclose on these points even when the disclosure is negative.

Sample size	For all statistical experiments, including kinetics analysis and conversion rate values were determined with n=3 as it is common practice in the field, exemplified in Nature, 527, 539-547 (2015) and J. Am. Chem. Soc. 141, 9964–9979 (2019).
Data exclusions	No data are excluded.
Replication	All attempts are performed independently and successfully replicated more than three times. We stated the number of replicates for each experiment in the paper. Furthermore, the reproducibility of the assays was confirmed by including appropriate positive and negative controls.
Randomization	This is not relevant to our study because this is the biochemical and structural analysis of biosynthetic enzymes. During these analysis, the products, catalyzed reaction, and structure of enzymes were not known.
Blinding	Blinding is not relevant for this study because we use enzymatically synthesized substrates and mutant enzymes for in vitro reactions. During these analysis, the products, catalyzed reaction, and structure of enzymes were not known. Further, corresponding structures characterized by NMR which is an unbiased technique.

Reporting for specific materials, systems and methods

We require information from authors about some types of materials, experimental systems and methods used in many studies. Here, indicate whether each material, system or method listed is relevant to your study. If you are not sure if a list item applies to your research, read the appropriate section before selecting a response.

Materials & experimental systems

n/a	Involved in the study
x	Antibodies
x	Eukaryotic cell lines
x	Palaeontology
x	Animals and other organisms
x	Human research participants
x	Clinical data

Methods

n/a	Involved in the study
x	ChIP-seq
x	Flow cytometry
x	MRI-based neuroimaging
